# Circadian activities of the brain MNK‐eIF4E signalling axis contribute to diurnal rhythms of some cognitive functions

**DOI:** 10.1111/ejn.15678

**Published:** 2022-05-07

**Authors:** Dong Liu, Jin Li, Hao Lin, Ethan Lorsung, Nam Le, Rubal Singla, Abhishek Mishra, Rikiro Fukunaga, Ruifeng Cao

**Affiliations:** ^1^ Department of Biomedical Sciences University of Minnesota Medical School Duluth MN USA; ^2^ Institute of Neuroscience and Translational Medicine, College of Life Science and Agronomy Zhoukou Normal University Zhoukou China; ^3^ Department of Biochemistry, Faculty of Pharmacy Osaka Medical and Pharmaceutical University Takatsuki Japan; ^4^ Department of Neuroscience University of Minnesota Medical School Minneapolis MN USA

**Keywords:** behaviour, circadian rhythm, cognition, eIF4E, MNK, mouse, phosphorylation

## Abstract

Although it is well recognized that the circadian timing system profoundly influences cognitive performance, the underlying molecular mechanisms remain poorly defined. Our previous work has found that the mitogen‐activated protein kinase‐interacting kinase (MNK)‐eukaryotic translation initiation factor 4E (eIF4E) axis, a conserved cellular signalling pathway regulating mRNA translation, modulates the function of the suprachiasmatic nucleus (SCN), the master circadian clock. Here, with the use of a combination of genetic, biochemical and behavioural approaches, we investigated the distribution and temporal regulation of eIF4E phosphorylation in the brain and its role in regulating the diurnal oscillations of some aspects of cognition in mice. We found that activities of the MNK‐eIF4E axis, as indicated by the level of eIF4E phosphorylation at Ser209, exhibited significant circadian oscillations in a variety of brain regions, including but not limited to the prefrontal cortex, the hippocampus, the amygdala and the cerebellum. Phosphorylated eIF4E was enriched in neurons but not in astrocytes or microglia. Mice lacking eIF4E phosphorylation (*eIF4E* ^*S209A/S209A*^) or the MNKs (*Mnk* ^1−/−,2−/−^), the kinases that phosphorylate eIF4E, exhibited impaired diurnal variations of novel object recognition, object location memory, Barnes maze learning and ambulatory activities. Together, these results suggest that circadian activities of the MNK‐eIF4E axis contribute to the diurnal rhythms of some cognitive functions, highlighting a role for rhythmic translational control in circadian regulation of cognitive performance.

## Graphical abstract

Liu et al. investigated the distribution and temporal regulation of eIF4E phosphorylation in the brain and its role in cognitive functions in mice. The results suggest that the daily rhythmic MNK‐eIF4E axis contributes to behavioural responses to novel objects and the diurnal rhythms in some forms of spatial memory.

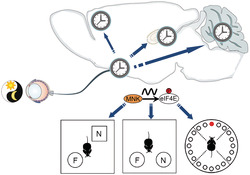

**Abbreviations**
Acb: nucleus accumbens
AIC: agranular insular cortex
BLA: basal amygdaloid nucleus
CC: cingulate cortex
CeA: central nucleus of the amygdala
CPu: caudate putamen
CxA: cortex‐amygdala transition area
DG: dentate gurus
DTT: dorsal tenia tecta
eIF4E: eukaryotic translation initiation factor 4E
En: endopiriform nucleus
GCL: granular cell layer
GFAP: glial fibrillary acidic protein
GP: globus pallidus
La: lateral amygdaloid nucleus
LOT: nucleus of the lateral olfactory tract
MAPK: mitogen‐activated protein kinase
MC: motor cortex
MNK: mitogen‐activated protein kinase‐interacting kinase
mPFC: medial prefrontal cortex
NOR: novel object recognition task
OLM: novel object location memory
Pir: piriform cortex
PSC: primary somatosensory cortex
PVA: paraventricular thalamic nucleus, anterior part
SCN: the suprachiasmatic nucleus

## 1 INTRODUCTION

Circadian (~24 h) rhythms are fundamentally important in regulating a variety of biochemical, physiological and behavioural processes in almost all living organisms (Silver & Kriegsfeld, 2014). The intrinsic property enables animals to temporally coordinate their life activities according to cyclic changes in the environment (Paul & Schwartz, 2007; Nitabach & Taghert, 2008), so that optimized physiological and metabolic efficiencies can be reached at the right time of a day (Dubruille & Emery, 2008; Karatsoreos & Silver, 2007; Martino & Harrington, 2020; Panda, 2016; Weil & Nelson, 2014). The circadian timing system is hierarchically organized in the brain. In mammals, the master circadian clock is located in the suprachiasmatic nucleus (SCN) of the hypothalamus (Reppert & Weaver, 2002). The SCN relays photic information from the retina to other brain regions to synchronize endogenous rhythms to ambient light/dark cycles (Cao et al., 2013; Mintz et al., 1999; Prosser et al., 1989; Tosini et al., 2012). Notably, circadian clocks also exist in a variety of extra‐SCN brain regions, as revealed by rhythmic expression of clock genes in these areas (Amir & Stewart, 2009).

Memory is the unifying power that holds our mental life together (Kandel et al., 2014). Although it is well recognized that the circadian timing system profoundly influences cognitive performance depending on the time of day (Gerstner & Yin, 2010), the underlying molecular mechanisms are not well understood. A newly formed memory remains in a dynamic or labile form for a short time, known as short‐term memory. Next, it is stored or consolidated into the physical organization of the brain, known as long‐term memory. The formation of long‐term memories requires neuronal activity‐induced de novo protein synthesis (Kandel et al., 2014). The new proteins contribute to structural changes (e.g., growth of existing or formation of new synapses) that are involved in memory storage. Recent advances obtained from genetic, physiological, pharmacological and biochemical studies provide strong evidence that mRNA translational control (control of protein synthesis) plays a key role in regulating long‐term changes in neural circuits and causes long‐term modifications in behaviour (Costa‐Mattioli et al., 2009).

Protein synthesis is primarily controlled at the step of translation initiation, which refers to the process by which the ribosome is recruited to the mRNA that culminates with the binding of the 80S ribosome to the mRNA initiation codon. This process starts with the recognition of the 5′ cap structure, m7GpppN (where N is any nucleotide) by the eukaryotic translation initiation factor 4E (eIF4E). As eIF4E is the least abundant initiation factor, 5′ cap‐recognition by eIF4E is rate‐limiting for translation and therefore a major target for regulation. The protein level and activities of eIF4E are controlled at different levels including transcription, phosphorylation and interactions with protein binding partners, such as the family of 4E binding proteins (Richter & Sonenberg, 2005). Importantly, eIF4E is a downstream target of the mitogen‐activated protein kinase (MAPK) signalling pathway, which is activated in response to extra‐ and intracellular stimuli, such as mitogens, growth factors and neuronal activities. MAPK activates the serine/threonine kinases MNK (mitogen‐activated protein kinase‐interacting kinase) 1 and MNK2 (Waskiewicz et al., 1997), which, in turn, phosphorylate eIF4E at a single amino acid, Ser209 (Furic et al., 2010; Ueda et al., 2004). The functional significance of eIF4E phosphorylation in mRNA translation remains incompletely understood (Proud, 2007). In our previous study (Gkogkas et al., 2014), we did not find a change in the rate of global protein synthesis in the brain of mice lacking eIF4E phosphorylation (*eIF4E* ^*S209A/S209A*^), indicating that eIF4E phosphorylation may regulate brain mRNA translation in a transcript specific manner.

We previously found that eIF4E phosphorylation plays a significant role in regulating the SCN circadian clock (Cao et al., 2015). The level of eIF4E phosphorylation exhibits strong rhythmicity in the SCN under constant conditions, which is critical to maintain robust clock gene oscillations in the SCN. Rhythmic oscillations of clock protein PERIOD1 and PERIOD2 are dampened in the SCN in the absence of eIF4E phosphorylation. Moreover, we found that light at night activates MNK, which, in turn, phosphorylates eIF4E. Rapid eIF4E phosphorylation after light exposure at night promotes light induced *Per1* and *Per2* mRNA translation and facilitates photic resetting of the SCN clock. Despite these findings, however, the physiological function of eIF4E phosphorylation in extra‐SCN brain clocks remains elusive. To start to pursue this problem, here, we investigated brain region‐ and cell type‐specific distribution of eIF4E phosphorylation in the mouse brain and its temporal regulation by the circadian clock. We also determined a role for eIF4E phosphorylation in regulating the diurnal oscillations of some aspects of cognition. Our results indicate that the rhythmic MNK‐eIF4E axis contributes to some diurnal rhythms and some aspects of cognition.

## 2 MATERIALS AND METHODS

### 2.1 Animals

The breeders of *eIF4E* ^*S209A/S209A*^ mice (RRID:MGI:4830312) on a C57BL/6J background were obtained from the laboratory of Nahum Sonenberg (Furic et al., 2010). C57BL/6J breeders were purchased from the Jackson Laboratory (Stock No: 000664; RRID:IMSR_JAX:000664). *Mnk* ^1−/−,2−/−^ mice (Ueda et al., 2004; RRID:MGI:3052698) were backcrossed to C57BL/6J mice for over 10 generations and used in the current study. Animals were housed in the animal facility at the University of Minnesota, Duluth under 12 h/12 h light/dark cycles with ad libitum access to mouse chow (LabDiet 5053) and tap water. The room temperature was maintained at 22 ± 1°C, and humidity was at 35–45%. All procedures were approved by the Institutional Animal Care and Use Committee at the University of Minnesota.

### 2.2 Mouse behavioral testing

Six to eight‐week‐old WT C57BL/6J, *eIF4E* ^*S209A/S209A*^ and *Mnk* ^1−/−,2−/−^ mice (male–female ratio at approximately 1:1) were used for all experiments. Naïve mice were used in each behavioural test, and mice were not reused for different tests. Different cohorts of mice were used for different time points. Seven to ten mice from each group were used for each behavioural test. Behavioural tests were performed at indicated time points under similar lighting conditions as the housing facility. Zeitgeber Time (ZT) 6 (6 h after light‐on) was used as ‘Day’ time point, and the white light intensity was 100 lux at cage level. ZT18 (6 h after light‐off) was used as the ‘Night’ time point, and dim red light was used (~10 lux at cage level). Mice were gently handled once a day for 3 days prior to the experiments to reduce stress during the behavioural tests. Animals were transferred to the testing room 30 min before experiments for habituation to the testing environment. Animals were assigned to groups based on their genotypes. For each genotype, mice were randomly assigned to the day (ZT 6) and night (ZT 18) groups using a random number table. No sample calculation was performed. Experimenters were blinded to genotypes during the testing and data analysis in the following tests that were performed.

#### 2.2.1 Novel object recognition (NOR) memory test

An NOR test was performed as described (Vogel‐Ciernia & Wood, 2014). Briefly, 24 h before training, the mouse was first placed in an open field arena (40 × 40 × 30 cm) without objects for habituation and allowed to freely explore the arena for 10 min. On the training day, two identical objects (glass bottles) were placed at the opposite corners (5 cm from the walls) of the arena. The mouse was then placed into the arena and allowed to freely explore the objects for 10 min before returning to the home cage. Twenty‐four hours after training, one object was replaced by a novel object (wooden cube). The mouse was returned to the arena to explore for 5 min. Mouse activities were video recorded by a high‐resolution camera (720p). Videos were inspected by blinded researchers and analysed by the ANY‐maze mouse tracking system (Stoelting Co. IL; RRID:SCR_014289). The accuracy and reliability of the ANY‐maze system have been validated with hand scoring in the pilot study. Time spent investigating novel or familiar object and total distance travelled in the arena were determined. Investigating was defined as the mouse placing its nose within a 2‐cm proximity of the object or touching the object with the nose or forepaws. Proportion of time spent investigating the novel or familiar object was calculated as Time_novel_ or Time_familiar_/(Time_novel_ + Time_familiar_). The discrimination index (DI) was calculated as (Time_novel_ − Time_familiar_)/(Time_novel_  Time_familiar_).

#### 2.2.2 Object location memory (OLM) test

An OLM test was performed as reported (Vogel‐Ciernia & Wood, 2014). Twenty‐four hours before training, the mouse was placed in an open field arena (40 × 40 × 30 cm) without objects for habituation for 10 min. On the training day, the mouse was exposed to two identical objects (glass bottles), which were placed at two specific locations in the arena (as in the NOR test). The mouse was allowed to explore the arena and both objects for 10 min and returned to the home cage. After 24 h, one object was placed in the same position, whereas the other object was moved to a novel location (the adjacent corner, 5 cm from the walls). The mouse was returned to the arena to explore for 5 min, and its behaviour was video recorded. Mouse behaviours were analysed by the ANY‐maze mouse tracking system to determine the time in investigating each object. Time spent investigating novel (N) or familiar (F) location and total distance travelled in the arena were determined. Proportion of time spent investigating objects at the novel or familiar location was calculated as Time_novel_ or Time_familiar_/(Time_novel_ + Time _familiar_). The DI was calculated as (Time_novel_ − Time_familiar_)/(Time_novel_ + Time_familiar)_.

#### 2.2.3 Barnes maze test

A Barnes maze test was performed as reported (Bach et al., 1995). The maze consisted of a white circular platform (92 cm in diameter) elevated at 95 cm from the floor with 20 equally spaced holes (5 cm in diameter) along the perimeter located at 2 cm from the edge of the platform. Visual cues were located on the walls of the room. A black escape box (20 × 9 × 9 cm) was placed under one hole to allow the escape of the mouse. In the habituation phase, mice were trained to identify and enter the escape box (1) by placement into the escape box for 2 min and (2) guidance to the escape box, where they remained for 2 min. In the following 4 days (the acquisition phase), mice were trained 2 trials/day starting at either ZT 6 or ZT 18 with an interval of 15 min between the trials. In each trial, a mouse was first briefly placed in a 10‐cm‐high cylindrical black start chamber at the centre of the maze and given 5 min to find the escape box. At the beginning of each session, a buzzer (80 dB) was sounded 1 min after mice were placed in the start chamber. After 10 s of the buzzer, the start chamber was lifted, and the mice were allowed to freely explore the maze to find the escape box for up to 5 min. Mice that failed to enter the escape box within 5 min were guided to the box and left in the box for 2 min before they were returned to their home cage. Mouse activities were video recorded throughout the session, and videos were analysed by the ANY‐maze software. The latency to find and enter the escape box was determined. One day after the training, a probe test was performed with the escape box removed. Mice were allowed to explore the maze for 90 s, and the time in four quadrants and travel distances were determined.

### 2.3 Brain harvesting and immunohistochemistry

Mice were sacrificed at indicated time points by cervical dislocation and decapitation. Six mice from each group were used for immunostaining. Brains were rapidly harvested, and brain tissue was processed for immunostaining as published (Cao et al., 2015). Briefly, brains were cut into 2‐mm coronal slices guided by an acrylic mouse brain slicer (Zivic instruments, Pittsburgh, PA), fixed in 4% paraformaldehyde for 6 hr at room temperature, and then transferred into 30% sucrose (w/v, with 2‐mM sodium azide and 3‐mM NaF) overnight at 4°C. Brain slices were thin cut (40 μm) using a sliding microtome (Leica SM2010R) and placed in phosphate‐buffered saline (PBS) containing 2‐mM sodium azide and 3‐mM NaF, pH 7.4. For the immunohistochemical staining, sections were first treated with .3% H_2_O_2_ and 20% methanol in PBS for 10 min to deactivate endogenous peroxidases and permeabilize the tissue and then blocked for 1 h in 10% goat serum/PBS and incubated (overnight, 4°C) in rabbit polyclonal anti‐phospho‐eIF4E (Ser209) antibody (1:1000 final dilution; Novus Biologicals, NBP1‐19923; RRID:AB_1641951). Next, tissue was incubated for 1.5 h at room temperature in biotinylated anti‐rabbit IgG (1:400; Vector Laboratories, Burlingame, CA; RRID:AB_2687893) and then placed in an avidin/biotin HRP complex for 1 h (prepared according to instructions of the manufacturer; Vector Laboratories; RRID:AB_2336819). Sections were washed in PBS (three times, 10 min per wash) between each labelling step. The signal was visualized using nickel‐intensified DAB substrate (Vector Laboratories; RRID:AB_2336382) and sections were mounted on gelatin‐coated slides with Permount media (SP15‐500, Fisher Scientific, Houston, TX).

The immunohistochemistry and immunofluorescent procedures were performed using brain sections from the same cohort of mice. For immunofluorescent labelling, tissue was permeabilized with PBST (PBS with 1% Triton X‐100) for 30 min, blocked as described above and then incubated (overnight, 4° C) in 5% goat serum/PBS with rabbit polyclonal anti‐phospho‐eIF4E (Ser209) antibody (1:300 final dilution; Novus Biologicals, NBP1‐19923; RRID:AB_1641951) and one of the following antibodies raised from mice: anti‐NeuN (1:300 final dilution; Millipore, MAB377; RRID:AB_2298772), anti‐GFAP (1:300 final dilution; Biolegend, 644701; RRID:AB_2109791) or anti‐CD11b (1:300 final dilution; Bio‐Rad, MCA711; RRID:AB_321292). The following day, sections were incubated (3 h, room temperature) in Alexa Fluor‐594‐conjugated goat anti‐rabbit IgG antibody (1:500; Molecular Probes, Eugene, OR; RRID:AB_142057) and/or Alexa Fluor‐488‐conjugated goat anti‐mouse IgG antibody (1:500; Molecular Probes, Eugene, OR; RRID:AB_143160). Brain sections were washed in PBS (three times, 10 min per wash) between each labelling step. Sections were mounted on slides with Cytoseal 60 (8310‐16, Richard‐Allan Scientific, Kalamazoo, MI). Bright‐field and fluorescent microscopic images were captured using a digital camera mounted on an inverted DMi8 Leica microscope (Nussloch, Germany). Confocal microscopy images were captured using a Zeiss 710 Meta confocal microscope (Oberkochen, Germany; RRID:SCR_018063). All confocal parameters (pinhole, contrast, brightness, etc.) were held constant for all data sets from the same experiment.

### 2.4 Brain harvesting and western blotting

Mice were sacrificed at indicated time points by cervical dislocation and decapitation. Three mice from each group were used for western blotting. Brains were rapidly harvested, and hippocampus, prefrontal cortex and cerebellum were dissected and frozen on dry ice. Tissue was homogenized with a pestle grinder (12‐141‐361, Fisher Scientific) and lysed using a lysis buffer, and western blotting analysis was performed as described (Cao et al., 2015). Briefly, brain lysates were electrophoresed into a 10% SDS‐PAGE gel, then transblotted onto polyvinylidene difluoride membranes (Immobilon‐P, Millipore; RRID:SCR_008983). Membranes were blocked in 10% skim milk (MP290288705, MP Biomedicals) and then incubated (overnight, 4°C) in PBST (with 5% BSA) with anti‐phospho‐eIF4E (Ser209) antibody (1:1000 final dilution; Novus Biologicals, NBP1‐19923; RRID:AB_1641951) or anti‐eIF4E antibody (1:1000, BD Transduction Laboratories, 610270; RRID:AB_397665). Next, membranes were incubated in PBST (with 5% skim milk) with an HRP‐conjugated secondary antibody (1:5000, GE Healthcare, donkey anti‐rabbit: NA931; donkey anti‐mouse: NA934). Between each antibody treatment, membranes were washed a minimum of three times (10 min/wash) in PBST. *Chemiluminescence* was developed using the Western Lightning Chemiluminescence Reagents (NEL103001EA, PerkinElmer) and detected on X‐ray films. Films were scanned into digital images and the density of the blots were determined using the Adobe Photoshop software (Adobe Systems Incorporated, San Jose, CA; RRID:SCR_014199).

### 2.5 Data analysis

For the p‐eIF4E intensity analysis, 10× grayscale microscopic images labelled for p‐eIF4E were obtained using a Leica DFC3000G camera. All imaging parameters (exposure time, light intensity, etc.) were held constant for all data sets from the same experiment. Three digital squares (size 50 × 50 pixels) were randomly placed in brain regions of interest, and the mean labelling intensity of the three squares was determined using Adobe Photoshop software (Adobe Systems Incorporated, San Jose, CA). A digital square (size 50 × 50 pixels) was then placed in a brain area where no p‐eIF4E was expressed to determine the intensity of non‐specific background staining. The background value was subtracted from the p‐eIF4E labelling value to obtain the normalized p‐eIF4E intensity. Three brain sections were used to obtain a mean value for each animal. Mean values from different animals were pooled into treatment groups and compared by one‐way analysis of variance (ANOVA) followed by SNK post‐tests using GraphPad Prism 9 (GraphPad Software, La Jolla, CA; RRID:SCR_002798). For the p‐eIF4E and NeuN colocalization assay, confocal microscopic images (40× magnification) of double labelling for p‐eIF4E and NeuN were collected. Individual cells were outlined based on DAPI staining, and the expression of p‐eIF4E (red), NeuN (green) or both (yellow) was determined based on densitometry values for red (p‐eIF4E) and green (NeuN) channels. The percentages of red, green or yellow cells were determined. A total of 23–41 cells were randomly chosen from high magnification images. Three images were used from each animal, and three WT mice were used for the analysis.

To compare the investigating time in NOR and OLM tests, two‐way ANOVA with post hoc Sidak's multiple comparison tests were applied. To compare DIs and distances traveled, two‐way ANOVA was used to compare effects of genotype among three groups, and Student's *t* test was used to compare day–night differences in each genotype. For acquisition and probe testing in Barnes maze, repeated measures two‐way ANOVA (RM ANOVA) with post hoc Sidak's multiple comparison tests were applied. *P* < .05 was accepted as statistically significant. Statistical analysis was performed, and graphs were analysed and plotted using GraphPad Prism 9 (GraphPad Software, La Jolla, CA; RRID:SCR_002798).

## 3 RESULTS

### 3.1 Circadian regulation of the activities of MNK‐eIF4E signalling axis in the brain

**FIGURE 1:**
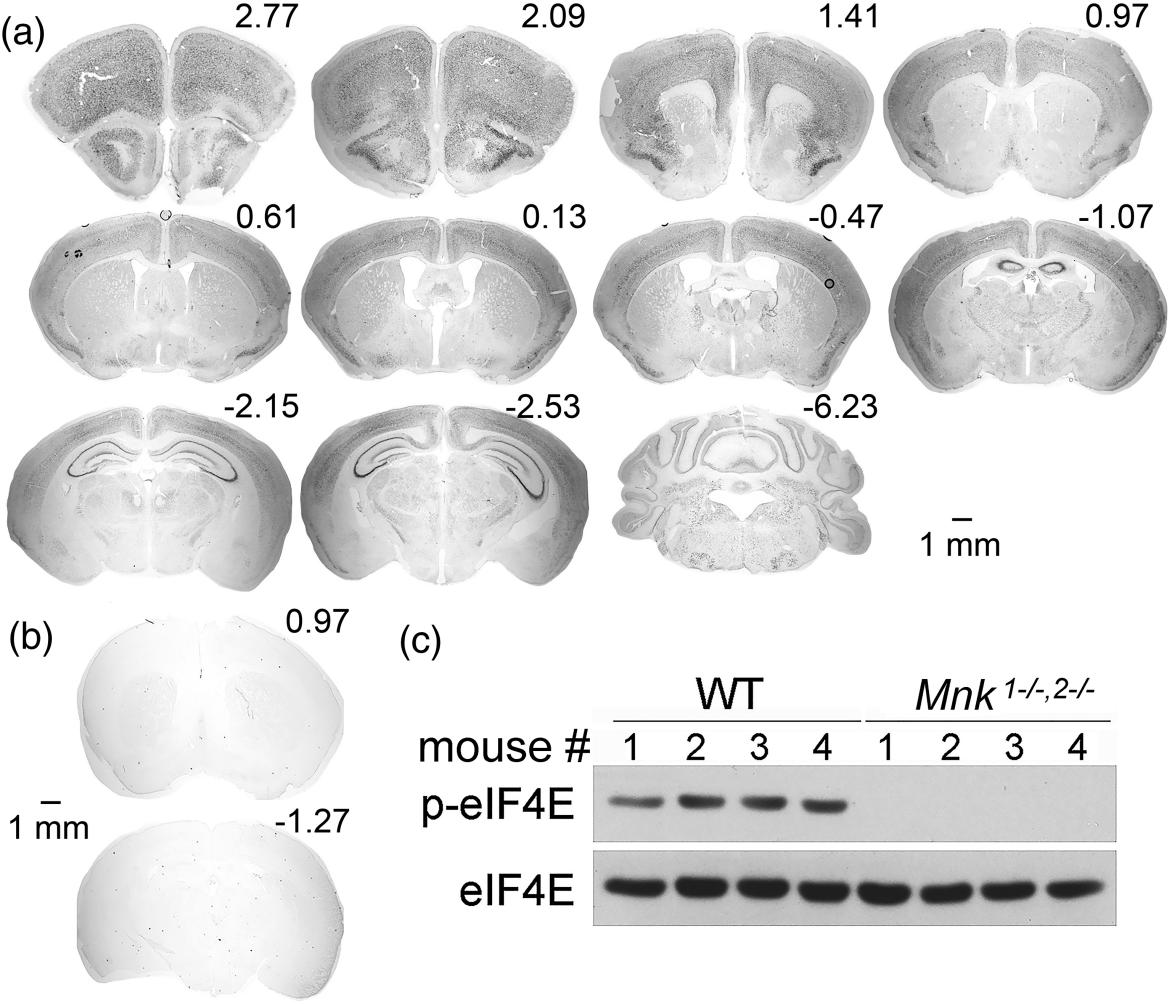
Phosphorylation of eIF4E in various brain regions is dependent on MNK1 and MNK2. (a) Representative coronal brain sections from wild type (WT) mice immunolabeled for phosphorylated eIF4E (Ser209). The coordinates (mm from bregma) of the brain sections are indicated at the upper right corner. Mice were sacrificed and brains were harvested at ZT6 (6 h after light‐on). Scale bar: 1 mm. (b) Representative coronal brain sections from *eIF4E* ^*S209A/S209A*^ mice immunolabeled for phosphorylated eIF4E (Ser209). No eIF4E phosphorylation was found in the brain of *eIF4E* ^*S209A/S209A*^ mice. Scale bar: 1 mm. (c) Western blotting indicates that eIF4E phosphorylation was eliminated in the brain of *Mnk* ^1−/−,2−/−^ mice. Each lane represents brain lysates from one animal

MNKs phosphorylate eIF4E at a single amino acid, Ser209. To determine the activities of MNK‐eIF4E axis, we first detected the expression of phosphorylated eIF4E (p‐eIF4E, Ser 209) in the brain by immunohistochemical staining. We found that p‐eIF4E was widely expressed in different brain regions of the wild type (WT) mice, including cerebral cortex, striatum, thalamus, hypothalamus, hippocampus, amygdala, cerebellum and pons (Figures 1a and S1 and S2). These brain regions are involved in a variety of brain functions, including learning and memory. eIF4E phosphorylation was not detected in the brain of *eIF4E* ^*S209A/S209A*^ mice, in which Ser209 of eIF4E is mutated to alanine and therefore cannot be phosphorylated, demonstrating the specificity of the anti‐p‐eIF4E antibody we used (Figure 1b). By western blotting, we confirmed that eIF4E phosphorylation was not present in the brain of *Mnk* ^1−/−,2−/−^ mice, in which both *Mnk1* and *Mnk2* are deleted, indicating that eIF4E is phosphorylated solely by MNK1 and MNK2 kinases in the brain (Figure 1c).

**FIGURE 2:**
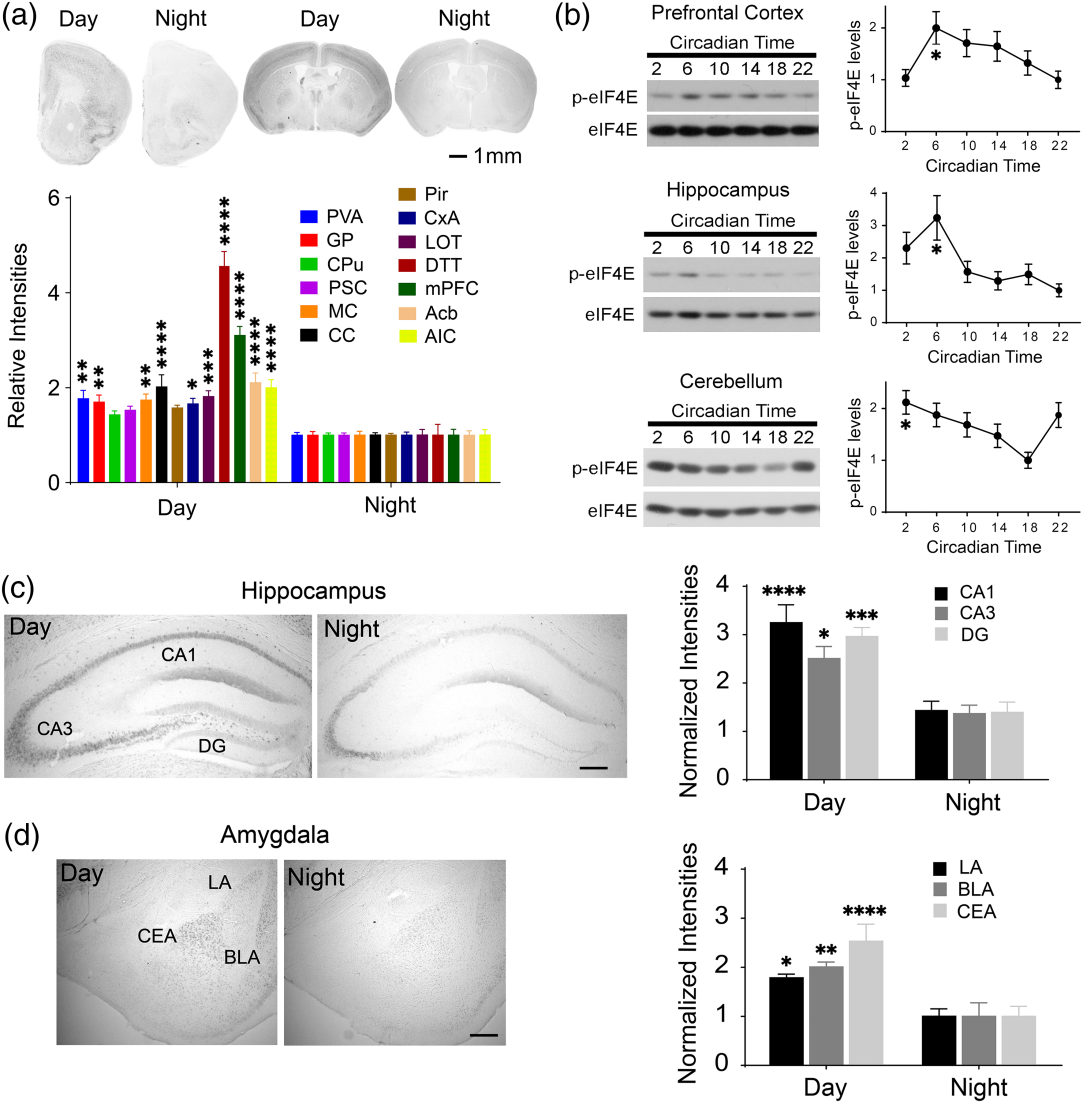
Diurnal oscillations of eIF4E phosphorylation in various mouse brain regions. (a) Top: representative microscopic images of coronal brain sections from wild type (WT) mice immunolabeled for phosphorylated eIF4E (Ser209). Scale bar: 1 mm. Bottom: quantitation of the staining intensities of p‐eIF4E in various brain regions. Mice were sacrificed, and brains were harvested during the day (ZT6) or at night (ZT18). *n* = 6 mice/group. The p‐eIF4E levels at night were normalized to be ‘1’ in respective brain areas. **P < .01, ***P < .001, ****P < .0001 versus Night. PVA, paraventricular thalamic nucleus, anterior part; GP, globus pallidus, CPu, striatum; PSC, primary somatosensory cortex; MC, motor cortex; CC, cingulate cortex; Pir, piriform cortex; CxA, cortex‐amygdala transition area; LOT, nucleus of the lateral olfactory tract; DTT, dorsal tenia tecta; mPFC, medial prefrontal cortex; Acb, nucleus accumbens; AIC, agranular insular cortex. (b) Circadian oscillations of eIF4E phosphorylation in the prefrontal cortex, hippocampus and cerebellum. Representative western blots are shown on the left. Quantitation of the blots is shown to the right. *n* = 3 mice/group. *P < .05 versus CT22 in the prefrontal cortex and hippocampus; *P < .05 vs CT18 in the cerebellum. (c) Diurnal variations in the level of eIF4E phosphorylation in the hippocampus. DG, dentate gyrus. *n* = 6 mice/group. **P < .01, ***P < .001, ****P < .0001 versus Night. Scale bar: 200 μm. (d) Diurnal variations in the level of eIF4E phosphorylation in the amygdala. CEA, central nucleus of the amygdala; LA, lateral amygdaloid nucleus; BLA, basal amygdaloid nucleus. *n* = 6 mice/group. *P < .05, **P < .01, ****P < .0001 versus Night. Scale bar: 200 μm

Next, to determine whether the level of eIF4E phosphorylation exhibits diurnal oscillations, mice were sacrificed, and brains were harvested in the middle of day (ZT, ZT6) or in the middle of night (ZT18). Immunostaining and quantitative analysis revealed significant day–night variations in the level of eIF4E phosphorylation in a wide variety of brain regions (*F*_(12, 130)_ = 18.69, *P* < .0001, two‐way ANOVA, Figure 2a), including paraventricular thalamic nucleus, anterior part (PVA), globus pallidus (GP), motor cortex (MC), cingulate cortex (CC), cortex‐amygdala transition area (CxA), nucleus of the lateral olfactory tract (LOT), dorsal tenia tecta (DTT), medial prefrontal cortex (mPFC), nucleus accumbens (Acb) and agranular insular cortex (AIC) (PVA: *t*_(130)_ = 3.892, *P* = .0021;GP: *t*_(130)_ = 3.514, *P* = .0079; MC: *t*_(130)_ = 3.707, *P* = .0040; CC: *t*_(130)_ = 5.111, *P* < .0001; CxA: *t*_(130)_ = 3.312, *P* = .0155; LOT: *t*_(130)_ = 4.086, *P* = .0010; DTT: *t*_(130)_ = 17.97, *P* < .0001; mPFC: *t*_(130)_ = 10.59, *P* < .0001; Acb: *t*_(130)_ = 5.59, *P* < .0001; AIC: *t*_(130)_ = 5.01, *P* < .0001; Sidak's multiple comparisons, Figure 2a). To determine whether the circadian clock or the light cycle regulates the level of eIF4E phosphorylation in the brain, by western blotting, we measured the level of eIF4E phosphorylation at six time points (Circadian Time, CT2, 6, 10, 14, 18 and 22) across a 24‐h cycle when mice were kept in constant darkness. We found significant circadian oscillations of eIF4E phosphorylation in the prefrontal cortex, hippocampus and cerebellum (PFC, *F*_(5, 12)_ = 2.937, *P* = .0397; hippocampus, *F*_(5, 12)_ = 3.909, *P* = .0246; cerebellum, *F*_(5, 12)_ = 3.196, *P* = .0460, one‐way ANOVA, Figures 2b and S3A), indicating that the diurnal oscillations of p‐eIF4E levels do not reply on the light–dark cycle. Interestingly, the level of eIF4E phosphorylation peaked in two forebrain structures prefrontal cortex and hippocampus at CT6, whereas the peak of eIF4E phosphorylation appeared in the cerebellum at CT22‐2, indicating significant phase differences of circadian eIF4E phosphorylation in different brain regions (Figure 2b). The levels of eIF4E were not different at different time points in these brain regions (PFC, *F*_(5, 12)_ = .3732, *P* = .8575; hippocampus, *F*_(5, 12)_ = 1.16, *P* = .3828; cerebellum, *F*_(5, 12)_ = 1.503, *P* = .2603, one‐way ANOVA, Figure S3B). Next, we further assessed the level of eIF4E phosphorylation at hippocampal CA1, CA3 and dentate gyrus (DG) areas during day (CT6) and night (CT18) by immunohistochemistry. We found significantly higher levels of eIF4E phosphorylation in all three hippocampal regions during the day as compared with the levels at night (*F*_(1, 27)_ = 54.2, *P* < .0001, two‐way ANOVA, CA1: *t*_(27)_ = 5.124, *P* < .0001; CA3: *t*_(27)_ = 3.214, *P* = .0101; DG: *t*_(27)_ = 4.413, *P* = .0004; Sidak's multiple comparisons, Figure 2c). Similarly, the levels of eIF4E phosphorylation were higher during the day in the lateral (LA), basolateral (BLA) and central (CEA) nuclei of the amygdala as compared with the levels at night (*F*_(1, 30)_ = 39.75, *P* < .0001, two‐way ANOVA, LA: *t*_(30)_ = 2.575, *P* = .0449; BLA: *t*_(30)_ = 3.309, *P* = .0073; CEA: *t*_(30)_ = 5.035, *P* < .0001, Sidak's multiple comparisons, Figure 2d).

### 3.2 eIF4E is preferentially phosphorylated in neurons but not in astrocytes and microglia

**FIGURE 3:**
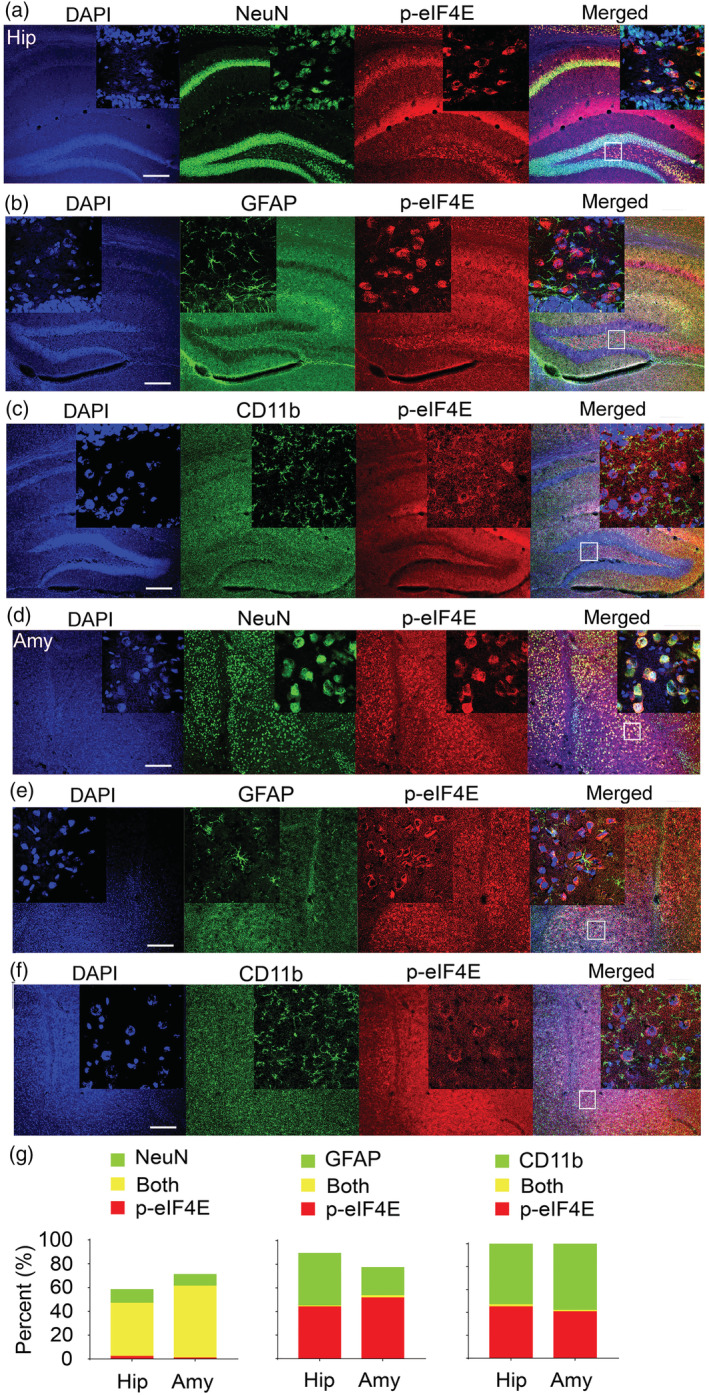
Cell specific eIF4E phosphorylation in the hippocampus and amygdala. (a–c) Representative confocal images of hippocampal coronal sections immunolabeled for p‐eIF4E (Ser209) (red) and (a) NeuN (green), a neuronal marker; (b) glial fibrillary acidic protein (GFAP, green), a marker for astrocytes; or (c) CD11b (green), a marker for microglia. Sections were counterstained by DAPI (blue), a cell nuclear dye. Framed regions are magnified and displayed in the insets. Scale bar: 100 μm. (d–f) Representative confocal microscopic images of coronal sections of the amygdala immunolabeled for phospho‐eIF4E (Ser209) (red) and (d) NeuN, (e) GFAP or (f) CD11b (green). Sections were counterstained by DAPI. Framed regions are magnified and displayed in the insets. Scale bar: 100 μm. (g) Percentages of cells expressing p‐eIF4E and NeuN (left), GFAP (middle) or CD11b (right) in the hippocampus (hip) and amygdala (Amy)

As eIF4E is widely phosphorylated in the brain, we next focused on the hippocampus and amygdala, two well‐defined brain regions important for learning and memory, to analyse cell‐type specific eIF4E phosphorylation. By double labelling for p‐eIF4E and NeuN, a neuronal marker, we found that phosphorylation of eIF4E was enriched in hippocampal pyramidal neurons in CA1, CA3 as well as in the granular cell layer (GCL) of the DG. We also found strong cellular colocalization of NeuN and p‐eIF4E expression in interneurons in the hilus of DG (Figure 3a), indicating that eIF4E is strongly phosphorylated in all hippocampal neurons. In contrast, the staining for GFAP, a marker for astrocytes, or CD11b, a marker for microglia, did not colocalize with the p‐eIF4E staining, indicating that eIF4E is preferentially phosphorylated in neurons but not in glial cells (Figure 3b,c). Similarly, in the amygdala, double labelling for NeuN and p‐eIF4E revealed strong cellular colocalization of NeuN and p‐eIF4E expression (Figure 3d). In contrast, labelling for GFAP or CD11b did not show colocalization with the p‐eIF4E labelling (Figure 3e,f). Quantitative analysis of cellular colocalization revealed that eIF4E was phosphorylated in ~80% of NeuN positive cells in the hippocampus and ~90% of NeuN positive cells in the amygdala but in less than 5% of GFAP or CD11b positive cells in both brain regions (Figure 3g), indicating that eIF4E phosphorylation is enriched in neurons but not in astrocytes or microglia in the hippocampus and amygdala.

### 3.3 Rhythmic eIF4E phosphorylation contributes to behavioural responses to novel objects and diurnal rhythms in novel object recognition

As eIF4E phosphorylation exhibited strong circadian rhythmicity in brain regions associated with learning and memory, including the hippocampus, the amygdala and the cerebral cortex (Figure 2), we next determined whether phosphorylation of eIF4E regulates NOR memory and its day–night variations. The NOR task is used to assess rodents' innate preference for novelty and relies on multiple brain regions. Before our main study, control experiments were performed to assess (A) the accuracy and reliability of ANY‐maze system as compared with hand scoring; (B) total exploration time for Objects 1 and 2; and (C) potential sex differences in the NOR and OLM tests. We found no difference between results obtained by ANY‐maze and by hand scoring (*F*_(1, 20)_ = .001076, *P* = .9742, Figure S4A). Naïve animals spent similar time exploring Object 1 (water bottle) and Object 2 (wooden cube) (*F*_(1, 30)_ = .058, *P* = .8113, Figure S4B). No difference was detected between male and female WT mice in DIs or total distance travelled in either test (NOR: *F*_(1,15)_ = .007, *P* = .933 for DI, *F*_(1,15)_ = .160, *P* = .695 for distance, OLM: *F*_(1,16)_ = .017, *P* = .897 for DI, *F*_(1,16)_ = .366, *P* = .554 for distance, Figure S4C,D). We therefore pooled data from male and female mice in the main study.

**FIGURE 4:**
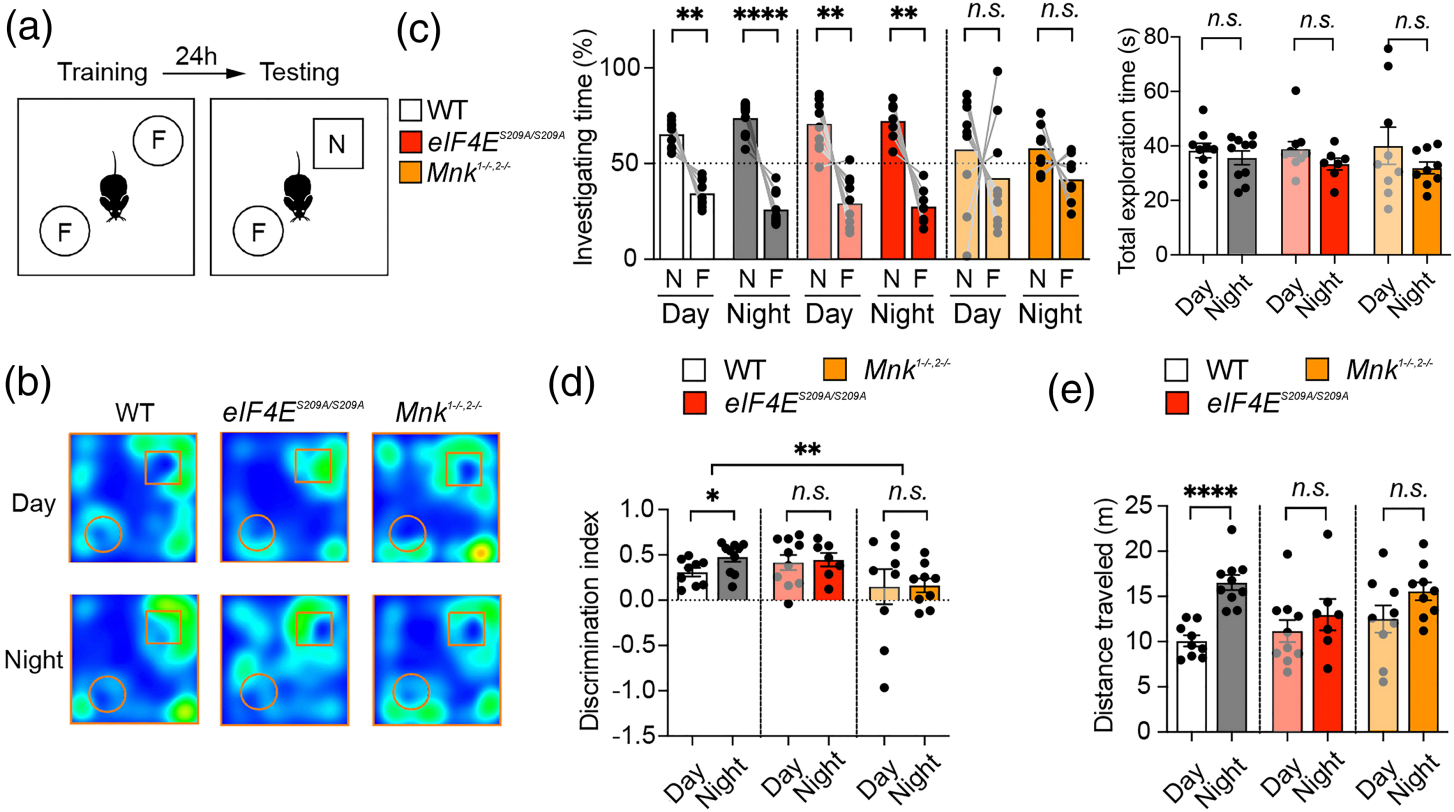
The MNK‐eIF4E axis contributes to responses to novel objects and diurnal rhythms in novel object recognition. (a) A schematic diagram of the novel object recognition (NOR) test. During the training session, mice were exposed to two familiar objects (F). After 24 h, one familiar object (F) was replaced by a novel object (N) in the testing session. Time animals spent investigating the two objects were measured. (b) Representative heatmaps exhibit time spent in investigating novel and familiar objects by the wild type (WT), *eIF4E* ^*S209A/S209A*^ and *Mnk* ^1−/−,2−/−^ mice during the day (ZT 6) or at night (ZT 18). (c) Bar graphs indicate the percentages of time spent in investigating novel or familiar objects and total exploration time in the NOR test. Grey lines indicate the paired values of the same mouse. (d) A bar graph indicates the discrimination index in the NOR test. The discrimination index was calculated as (Time_novel_ − Time_familiar_)/Time_total_. (e) A bar graph indicates the distance travelled in the NOR test. *N* = 7–10 mice/group. All data in (c)–(e) are presented as individual values and mean ± SEM. *P < .05, **P < .01, ****P < .0001, *n.s*., not significant

We first compared NOR memory during the day (ZT6) and at night (ZT18) in WT, *eIF4E* ^*S209A/S209A*^ and *Mnk* ^1−/−,2−/−^ mice (Figure 4a). The WT mice exhibited a strong preference for a novel object over a familiar object both during the day and at night, as indicated by spending more time investigating the novel over the familiar object (day: *t*_(8)_ = 6.50, *P* = .001; night: *t*_(9)_ = 8.87, *P* < .0001, Sidak's multiple comparisons, Figure 4b,c). The total exploration time was ~35 s, and it was not different between day and night or among mice of different genotypes (*F*_(1.6, 37.9)_ = .29, *P* = .696, two‐way ANOVA, Figure 4c). The DI [(Time_novel_ − Time_familiar_)/(Time_novel_ + Time_familiar_)] was moderately but significantly higher at night compared with during the day in the WT mice (*t*_(17)_ = 2.31, *P* = .035, Figure 4d). The *eIF4E* ^*S209A/S209A*^ mice exhibited intact preference for a novel object during the day and at night (day: *t*_(9)_ = 4.96, *P* = .005; night: *t*_(6)_ = 6.00, *P* = .006, Sidak's multiple comparisons, Figure 4b,c). However, no significant difference in DI was detected in the *eIF4E* ^*S209A/S209A*^ mice between day and night (*t*_(15)_ = .26, *P* = .796, Figure 4d), indicating that the NOR memory is intact, but day–night oscillations are impaired in the *eIF4E* ^*S209A/S209A*^ mice. The *Mnk* ^1−/−,2−/−^ mice exhibited impaired preference for a novel object both during the day and at night (day: *t*_(8)_ = .78, *P* = .976; night: *t*_(8)_ = 2.13, *P* = .333, Sidak's multiple comparisons, Figure 4b,c). The DIs were not different between day and night in the *Mnk* ^1−/−,2−/−^ mice (*t*_(16)_ = .07, *P* = .946) and were significantly decreased compared with the WT mice (*F*_(1.5, 13.4)_ = 4.31, *P* = .045, two‐way ANOVA, Figure 4d), indicating that the diurnal oscillation of NOR memory is also impaired in the *Mnk* ^1−/−,2−/−^ mice. Lastly, the WT mice exhibited a day–night difference in total distance travelled during the NOR test (*t*_(17)_ = 6.10, *P* < .0001), but neither the *eIF4E* ^*S209A/S209A*^ (*t*_(15)_ = .88, *P* = .392) nor the *Mnk* ^1−/−,2−/−^ mice (*t*_(16)_ = 1.70, *P* = .109) exhibited such day–night differences (*F*_(1,9)_ = 24.25, *P* = .0008, two‐way ANOVA, Figure 4e), indicating impaired diurnal oscillations of ambulatory activities in these mice. Together, these results suggest that the MNK‐eIF4E axis contributes to novel object preference and to diurnal rhythmicity in object recognition.

**FIGURE 5:**
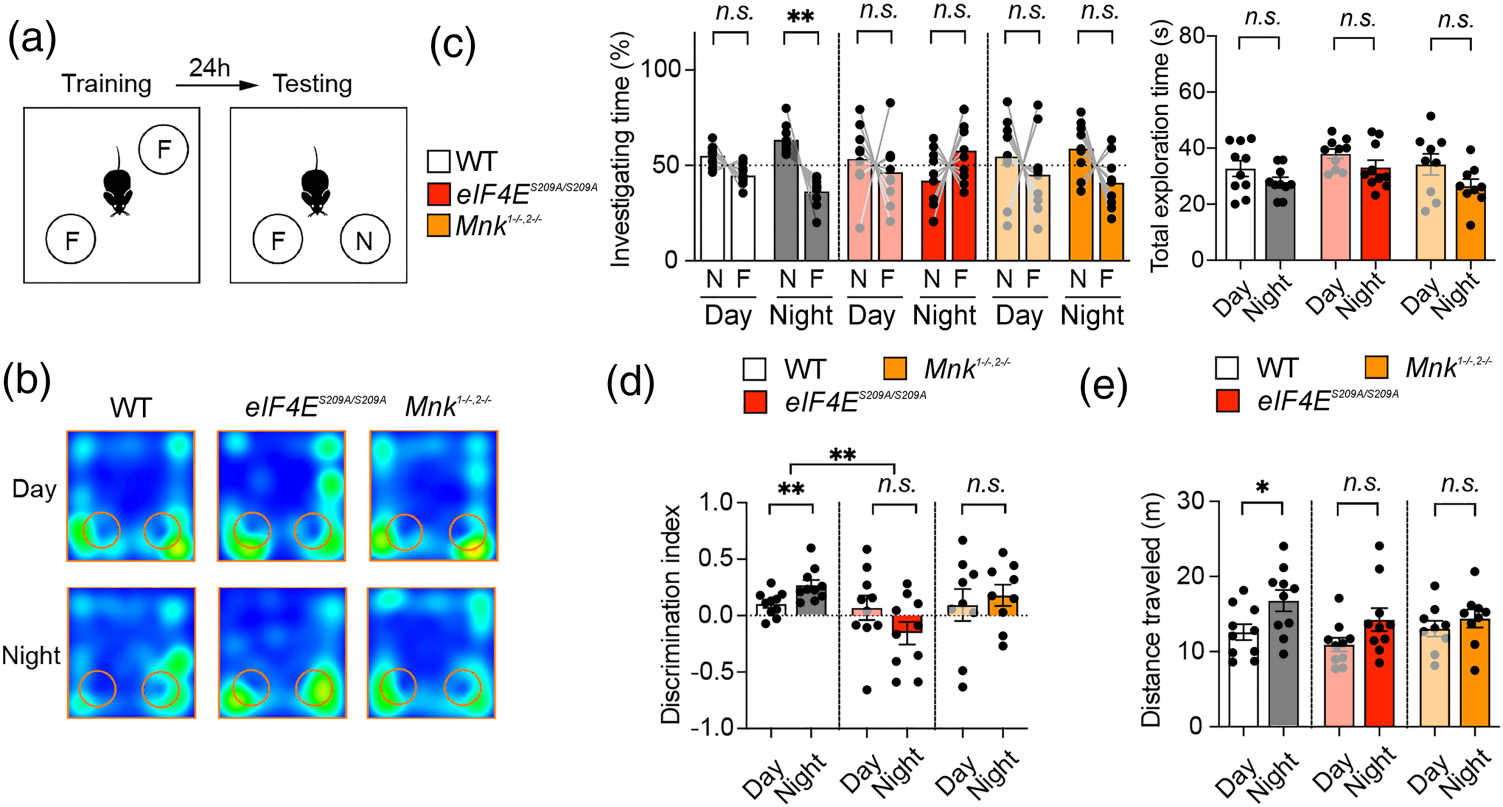
The MNK‐eIF4E axis contributes to responses to novel object locations and diurnal rhythms in spatial memory. (a) A schematic diagram of the object location memory (OLM) test. During the training session, mice were exposed to two identical objects (F). After 24 h, one object was left at the same familiar location (F), and the other object was moved to a novel location (N). Time animals spent investigating the two objects was measured. (b) Representative heatmaps exhibit time spent in investigating the objects at the novel and familiar location by the wild type (WT), *eIF4E* ^*S209A/S209A*^ and *Mnk* ^1−/−,2−/−^ mice in the OLM test performed during the day (ZT 6) or at night (ZT 18). (c) Bar graphs indicate the percentages of time spent in investigating the novel or familiar objects and the total exploration time in the OLM test. Grey lines indicate the paired values of the same mouse. (d) A bar graph indicates the discrimination index in the OLM test. The discrimination index was calculated as (Time_novel_ − Time_familiar_)/Time_total_. (e) A bar graph indicates the distance travelled in the OLM test. *N* = 9–10 mice/group. All data in (c)–(e) are presented as individual values and mean ± SEM. *P < .05, **P < .01, ***P < .001, *n.s*., not significant

To complement the NOR test, we performed a novel OLM test using a different cohort of animals (Figure 5a). NOR tests non‐spatial memory of object identity, whereas OLM tests spatial memory and highly relies on the hippocampus. We detected significant preference towards the object placed at a novel location at night but not during the day in the WT mice (day: *t*_(9)_ = 3.03, *P* = .083; night: *t*_(9)_ = 5.82, *P* = .002, Sidak's multiple comparisons, Figure 5b,c). Strikingly, neither the *eIF4E* ^*S209A/S209A*^ (day: *t*_(9)_ = .64, *P* = .990; night: *t*_(9)_ = 1.56, *P* = .633) nor the *Mnk* ^1−/−,2−/−^ mice (day: *t*_(8)_ = .66, *P* = .989; night: *t*_(8)_ = 1.92, *P* = .437) exhibited significant preferences towards the object at a novel location during the day or at night (Figure 5b,c). The total exploration time was ~35 s, and it was not different between day and night or among mice of different genotypes (*F*_(1.2, 9.9)_ = .21, *P* = .709, two‐way ANOVA, Figure 5c). The DI was higher at night than during the day in the WT mice (*t*_(18)_ = 2.89, *P* = .0098) but was not different between day and night in either the *eIF4E* ^*S209A/S209A*^ (*t*_(18)_ = 1.53, *P* = .144) or the *Mnk* ^1−/−,2−/−^ mice (*t*_(16)_ = .50, *P* = .621, Figure 5d). The DIs in the *eIF4E* ^*S209A/S209A*^ were significantly decreased compared with the WT mice (*t*_(8)_ = 6.58, *P* = .014, *F*_(2,18)_ = 3.77, *P* = .043, two‐way ANOVA, Figure 5d). Consistent with the results from the NOR test, the WT animals exhibited a day–night difference in the total distance travelled during the test (*t*_(18)_ = 2.37, *P* = .029), but the day–night difference was diminished in the *eIF4E* ^*S209A/S209A*^ (*t*_(18)_ = 1.89, *P* = .076) and the *Mnk* ^1−/−,2−/−^ mice (*t*_(16)_ = .85, *P* = .410, Figure 5e). Together, these results suggest that the MNK‐eIF4E axis contributes to responses to novel object locations and diurnal rhythms in spatial memory.

### 3.4 Rhythmic eIF4E phosphorylation contributes to diurnal rhythms during acquisition but not in memory assessments in Barnes maze task

**FIGURE 6:**
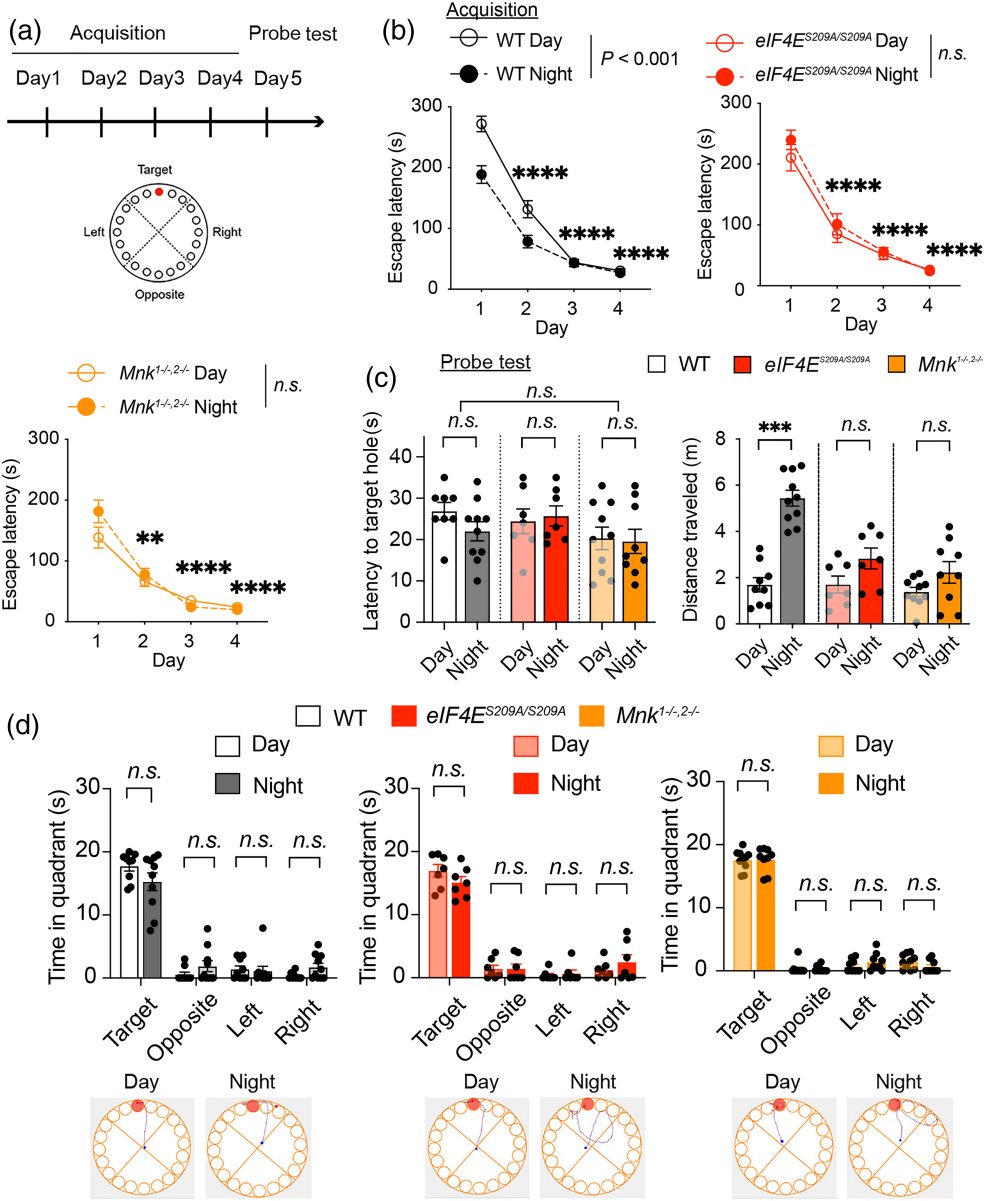
The MNK‐eIF4E axis contributes to diurnal rhythms during acquisition but not memory assessment in Barnes maze task. (a) A time‐line diagram of the Barnes maze test. In the acquisition phase, mice were trained 2 trials/day for 4 consecutive days starting at either ZT 6 or ZT 18. One day after the acquisition phase, a probe test was performed at the same time when training was performed. A schematic diagram below illustrates the set‐up of a Barnes maze. (b) Line graphs indicate the escape latency in each day of the acquisition phase performed during the day (ZT6) or at night (ZT18). **P < .01, ***P < .001, ****P < .0001 versus Day 1, *n.s*., not significant. *n* = 7–10 mice/group. Data are presented as mean ± SEM. Note that the escape latency was shorter at night compared with during the day in the wild type (WT) but not in the *eIF4E* ^*S209A/S209A*^ or *Mnk* ^1−/−,2−/−^ mice. (c) Left: Bar graph indicates the escape latency in the probe test performed during the day (ZT6) or at night (ZT18). Right: Bar graph indicates the distance traveled in the probe test performed during the day (ZT6) or at night (ZT18). Note that the day–night variations in the total distance traveled were diminished in the *eIF4E* ^*S209A/S209A*^ or *Mnk* ^1−/−,2−/−^ mice. ***P < .001, *n.s*., not significant. *n* = 7–10 mice/group. Data are presented as individual values and mean ± SEM. (d) Top: bar graphs indicating time spent in each quadrant in the first 20 s of the probe test. Bottom: representative images indicating the escaping routes in the first 20 s of the probe test

To further investigate the regulation of spatial learning and memory by eIF4E phosphorylation, we performed a Barnes maze test. It is also a hippocampal‐dependent task where animals learn the relationship between distal cues in the surrounding environment and a fixed escape location (Bach et al., 1995). The test consists of training in four consecutive days (the acquisition phase) and a probe test on the fifth day (Figure 6a). During the acquisition phase, the WT mice demonstrated significant daily improvements in finding the escape location as exhibited by decreased escape latency over the four training days both during the day (ZT6) (Day 2 vs. Day 1: *t*_(15)_ = 10.98, *P* < .0001; Day 3 vs. Day 1: *t*_(15)_ = 16.55, *P* < .0001; Day 4 vs. Day 1: *t*_(15)_ = 18.58, *P* < .0001) and at night (ZT18) (Day 2 vs. Day 1: *t*_(19)_ = 7.43, *P* < .0001; Day 3 vs. Day 1: *t*_(19)_ = 10.97, *P* < .0001; Day 4 vs. Day 1: *t*_(19)_ = 10.97, *P* < .0001, Figure 6b). When trained at night, the WT mice exhibited decreased escape latency compared with when trained during the day (*F*_(1, 34)_ = 15.11, *P* = .0004, two‐way ANOVA). Although the *eIF4E* ^*S209A/S209A*^ or *Mnk* ^1−/−,2−/−^ mice also learned and improved their performance significantly over the 4 days (*eIF4E* ^*S209A/S209A*^ Day: Day 2 vs. Day 1: *t*_(13)_ = 7.12, *P* < .0001; Day 3 vs. Day 1: *t*_(13)_ = 7.28, *P* < .0001; Day 4 vs. Day 1: *t*_(13)_ = 8.44, *P* < .0001; *eIF4E* ^*S209A/S209A*^ Night: Day 2 vs. Day 1: *t*_(13)_ = 6.73, *P* < .0001; Day 3 vs. Day 1: *t*_(13)_ = 10.55, *P* < .0001; Day 4 vs. Day 1: *t*_(13)_ = 12.94, *P* < .0001; *Mnk* ^1−/−,2−/−^ Day: Day 2 vs. Day 1: *t*_(17)_ = 4.11, *P* = .004; Day 3 vs. Day 1: *t*_(17)_ = 6.05, *P* < .0001; Day 4 vs. Day 1: *t*_(17)_ = 6.48, *P* < .0001; *Mnk* ^1−/−,2−/−^ Night: Day 2 vs. Day 1: *t*_(17)_ = 5.02, *P* = .0006; Day 3 vs. Day 1: *t*_(17)_ = 9.03, *P* < .0001; Day 4 vs. Day 1: *t*_(17)_ = 9.33, *P* < .0001) in the acquisition phase, the escape latency was not significantly different when trained during the day compared with when trained at night in either the *eIF4E* ^*S209A/S209A*^ or the *Mnk* ^1−/−,2−/−^ mice (*eIF4E* ^*S209A/S209A*^: *F*_(1, 34)_ = 1.52, *P* = .227; *Mnk* ^1−/−,2−/−^: *F*_(1, 26)_ = 1.19, *P* = .285, two‐way ANOVA, Figure 6b), indicating diminished day–night differences in spatial learning in these mice. In the probe test, we did not detect any differences in the latency to reach the target hole between day and night and in mice of any genotype, and there was no difference in escape latency between mice of different genotypes (*F*_(1, 9)_ = .57, *P* = .471, two‐way ANOVA, Figure 6c). However, the total distance travelled was longer at night compared with that during the day in the WT mice (*t*_(8)_ = 7.83, *P* = .0002), and such day–night difference was diminished in the *eIF4E* ^*S209A/S209A*^ and the *Mnk* ^1−/−,2−/−^ mice (*eIF4E* ^*S209A/S209A*^: *t*_(6)_ = 2.46, *P* = .141; *Mnk* ^1−/−,2−/−^: *t*_(8)_ = 1.71, *P* = .334, Figure 6c). As almost all mice moved straightly from the start chamber to the target hole within the first 30 s of the probe test, we analysed the time in quadrant in the first 20 s during the probe test. No different was found between day and night in all genotypes (WT: *F*_(1,17)_ = .02, *P* = .877; *eIF4E* ^*S209A/S209A*^: *F*_(1,12)_ = 3.40, *P* = .09; *Mnk* ^1−/−,2−/−^: *F*_(1,17)_ = 2.42, *P* = .138, two‐way ANOVA, Figure 6d). Together, these data indicate that the MNK‐eIF4E axis contributes to the day–night variations of some aspects of cognitive performance in the Barnes maze test.

## 4 DISCUSSION

In the current study, we investigated brain‐wide distribution of eIF4E phosphorylation and its temporal regulation by the circadian clock. We further assessed a role for eIF4E phosphorylation in the diurnal oscillations of some cognitive tests using NOR, OLM and Barnes maze tests. We found that the activities of the MNK‐eIF4E axis, as indicated by the level of eIF4E phosphorylation at Ser209, exhibited significant diurnal oscillations in a variety of brain regions. Phosphorylated eIF4E was enriched in neurons but not in astrocytes and microglia. Significant day–night differences in NOR and OLM memory, Barnes spatial learning and ambulatory activities were observed in the WT mice but not in the *eIF4E* ^*S209A/S209A*^ and *Mnk* ^1−/−,2−/−^ mice. In addition, *Mnk* ^1−/−,2−/−^ mice exhibited impaired behavioural responses to novel objects, and both *eIF4E* ^*S209A/S209A*^ and *Mnk* ^1−/−,2−/−^ mice exhibited impaired behavioural responses to novel object locations. These results suggest that the rhythmic activities of brain‐wide MNK‐eIF4E axis contribute to the diurnal rhythms of some aspects of cognitive performance.

Although it is recognized that circadian clocks also exist in a variety of extra‐SCN brain regions, their functional significance remains to be fully understood. These brain regions, including the prefrontal cortex, hippocampus, amygdala and cerebellum, modulate a variety of neural processes including learning and memory, social behaviours, anxiety and addiction. Consistent with this, a variety of neurophysiological processes are rhythmically regulated by the circadian clock, which accounts for the time‐of‐day variations in our sensory, motor, learning, memory and social functions (Amir & Stewart, 2009). Despite intensive studies on the neurophysiology of the SCN clock, however, little is known on how circadian clocks in other brain regions function to regulate rhythmic brain physiology and whether their dysregulation is involved in pathogenesis of various brain diseases. It is therefore important to understand the molecular mechanisms in the extra‐SCN circadian clocks in the brain.

We were promoted to study temporal regulation of eIF4E phosphorylation and its role in regulating rhythmic brain activities by our findings that eIF4E phosphorylation regulates autonomous circadian oscillations and photic entrainment of the SCN circadian clock (Cao et al., 2015). As eIF4E phosphorylation plays a fundamental role in translational control in the SCN, we asked whether it may also play a significant role in extra‐SCN brain regions. Interestingly, we found widespread phosphorylation of eIF4E in many brain regions, which are associated with a variety of brain functions. At the cellular level, we found phosphorylation of eIF4E is enriched in neurons, suggesting it may regulate neuron‐specific translational control programme. Indeed, a study found that BDNF stimulation of protein synthesis in cortical neurons requires MNK1 and eIF4E phosphorylation (Genheden et al., 2015). Precise regulation of the level of eIF4E phosphorylation appears to be important for normal brain function. Hyperphosphorylation of eIF4E was implicated in brain diseases such as fragile X syndrome and its mouse model, the *Fmr1* ^*−/y*^ mice (Gkogkas et al., 2014). By rectifying the level of eIF4E phosphorylation, core behavioural deficits, synaptic plasticity alterations and dendritic spine morphology defects are rescued in the fragile X mice. On the contrary, loss of eIF4E phosphorylation has been linked to depressive‐like behaviours in mice by two recent studies (Aguilar‐Valles et al., 2018; Amorim et al., 2018). In the peripheral nervous system, the MNK‐eIF4E axis also contributes to injury‐induced nociceptive plasticity and the development of chronic pain (Moy et al., 2017).

The circadian timing system profoundly influences cognitive performance in animals. Previous studies in multiple organisms revealed that circadian rhythmicity is involved in the formation, stability and recall of memories (Gerstner & Yin, 2010). Time‐of‐day effects on neurophysiology and memory have been reported (Krishnan & Lyons, 2015). Genetic disruption of the core clock genes impairs hippocampus‐dependent memory (Wardlaw et al., 2014). For example, *Bmal1* ^−/−^ mice exhibit impaired hippocampal long‐term potentiation (LTP), contextual fear and spatial memory. However, most studies show that SCN lesions do not produce memory deficits (reviewed in Ruby, 2021). SCN lesion in hamsters has no effect on object recognition, spontaneous alteration, passive avoidance or conditioned place avoidance (Cain & Ralph, 2009; Cain et al., 2012; Fernandez et al., 2014). SCN lesion in mice impairs contextual fear memory and spatial memory (Phan et al., 2011). In the disruptive phase shift (DPS) model, where circadian rhythm in the SCN is disrupted by shifted ambient lighting in hamsters, severe deficits in object recognition and spatial memory are found but LTP in hippocampal DG and CA1 remains intact (McMartin et al., 2021). Interestingly, SCN lesion can rescue these memory deficits in the DPS hamsters (Fernandez et al., 2014).

The hippocampus is important for long‐term memory formation. Interestingly, prominent circadian rhythmicity in neuronal activities has been found in the hippocampus, corresponding to daily fluctuation in learning and memory (Gerstner & Yin, 2010; Smarr et al., 2014). In the hippocampus, LTP, a cellular model for synaptic plasticity has been shown to change depending on the time of day in mice. In slice recordings of CA1, LTP induced during the subjective night results in an increase in amplitude of the population spikes with a slower decay of field EPSPs, relative to subjective day induction (Chaudhury et al., 2005). Interestingly, when hippocampal slices are harvested during the light or dark phase and then recorded in the opposite phase, the induction of LTP follows that of the light cycle rather than that of the circadian phase at the time the tissue was harvested (Chaudhury et al., 2005). This result suggests that the hippocampal clock oscillates in a circadian manner, independent of the SCN, providing a local mechanism to drive daily changes in LTP, which indicates functional changes in hippocampal dependent memory based on the time of day. Targeted deletion of *Bmal1* in the forebrain excitatory neurons (including the hippocampal pyramidal neurons but sparing the SCN neurons) leads to deficits in the Barnes maze test and time‐of‐day dependent NOR memory, also suggesting that peripheral brain clocks can regulate oscillations of memory performance independent of the SCN central clock (Snider et al., 2016).

Circadian oscillations of clock gene expression and MAPK activities are found in the hippocampus (Dolci et al., 2003; Eckel‐Mahan et al., 2012; Wang et al., 2009). The cyclic activation of hippocampal ERK MAPK signalling, the upstream pathway that leads to eIF4E phosphorylation, has been suggested to be involved in the persistence of long‐term memories (Eckel‐Mahan et al., 2008). SCN lesion abolishes the MAPK rhythms in the hippocampus (Phan et al., 2011). Here, we found the level of eIF4E phosphorylation also demonstrates strong daily oscillations in the hippocampus when animals were kept in constant conditions: higher in the day and lower at night. It reached a peak at CT6 and a nadir at CT 22, consistent with the rhythms of the ERK MAPK signalling activities in the hippocampus (Eckel‐Mahan et al., 2008). These data indicate that the eIF4E phosphorylation may be part of the clock regulated signalling mechanisms that are involved in the daily rhythmic hippocampal physiology. However, as the NOR task performance is low in the morning and high at night, whereas it is the opposite for contextual fear conditioning (Chaudhury & Colwell, 2002; Ruby, 2021), the circadian regulation of memory cannot be explained by a single oscillating molecule or process. In our study, as the *eIF4E* ^*S209A/S209A*^ and *Mnk* ^1−/−,2−/−^ mice are global mutants, we do not really address a specific role of hippocampus or any other specific brain regions. Further studies are required to define a specific role for the hippocampal clock and its output signalling pathways in cognitive regulation.

To access a role for eIF4E phosphorylation in rhythmic neurophysiological functions, we studied potential changes in different tests including NOR, OLM and Barnes maze tests using the two mouse models without eIF4E phosphorylation, the *eIF4E* ^*S209A/S209A*^ and *Mnk* ^1−/−,2−/−^ mice. Overall, our results demonstrate consistent diurnal variations in NOR, OLM and Barnes maze learning, as indicated by significant day–night differences in all these tests in WT mice. Mice performed better at night compared with during the day. These results are not surprising given that mice are nocturnal animals and are more active at night than during the day. It is worth mentioning that the lighting conditions were different for day and night behavioural tests in the current study. The light intensity of 100 lux during the day is sufficient to influence behavioural activities in mice. Therefore, we cannot rule out the possibility that the day/night differences in the results were due to the different lighting conditions rather than circadian changes. Significantly, our study further demonstrates consistent impairments of diurnal variations in some aspects of cognitive performances by all three tests in *eIF4E* ^*S209A/S209A*^ and *Mnk* ^1−/−,2−/−^ mice, which indicate the day–night difference in NOR, OLM and Barnes maze learning is modulated by rhythmic eIF4E phosphorylation. Notably, our Barnes maze paradigm was not able to detect significant day–night differences in Barnes maze memory in mice of all genotypes. This could be due to a ‘floor effect’. In the probe test, almost all mice moved straightly from the start chamber to the target hole within the first 30 s. Our testing paradigm may be too easy for the mice to reveal potential differences between day and night and between WT and mutants. Interpreting these results, however, is difficult in part due to the lack of significant preferences for the novel objects in NOR and/or OLM tasks in *eIF4E* ^*S209A/S209A*^ and *Mnk* ^1−/−,2−/−^ mice. There is also a lack of significant difference between WT and *eIF4E* ^*S209A/S209A*^ or *Mnk* ^1−/−,2−/−^ mice in Barnes maze memory. Another complication to a simple interpretation of the Barnes maze results is that the lack of diurnal difference in the acquisition phase in the *eIF4E* ^*S209A/S209A*^ and *Mnk* ^1−/−,2−/−^ mice appears to be due to improved daytime acquisition compared to WT mice rather than deficits.

In summary, our study identifies the MNK‐eIF4E axis as a circadian regulator in different brain regions, which may be important for some aspects of time‐of‐day specific cognitive functions by controlling neuronal mRNA translation. Although the translational targets of eF4E phosphorylation in specific brain regions remain to be identified in future studies, our findings presented here will advance our understanding of the signalling mechanisms in the extra‐SCN circadian clocks in the brain. *P*otential therapeutics targeting the MNK‐eIF4E axis may be developed to treat circadian and sleep problems that are associated with a variety of neurological and psychiatric diseases.

## CONFLICT OF INTEREST

The authors declare no conflicts of interest.

## AUTHOR CONTRIBUTIONS

R.C. designed the research. D.L., J.L., H.L., N.L., R.S., A.M. and R.C. performed experiments and analysed data. R.F. and R.C. contributed key research resources. D.L., J.L., E.L. and R.C. wrote the paper.

### PEER REVIEW

The peer review history for this article is available at https://publons.com/publon/10.1111/ejn.15678.

## Supporting information


**Figure S1** **(Related to Figure 1). Representative microscopic images indicating expression of phospho‐eIF4E in a coronal brain section.** The following brain regions are magnified and displayed separately: MC (motor cortex), AIC (agranular insular cortex), mPFC (medial prefrontal cortex), En (endopiriform nucleus), DTT (dorsal tenia tecta), and Pir (piriform cortex). Scale bar in magnified images: 200 μm.
**Figure S2** **(Related to Figure 1). Representative microscopic images indicating expression of phospho‐eIF4E in a coronal brain section.** The following brain regions are magnified and displayed separately: MC (motor cortex), PSC (primary somatosensory cortex), Pir (piriform cortex), LOT (nucleus of the lateral olfactory tract), PVA (paraventricular thalamic nucleus, anterior part), GP (globus pallidus), CC (cingulate cortex), and CPu (caudate putamen). Scale bar: 200 μm.
**Figure S3.** Western blotting images. A. Full length western blotting images of the blots shown in Figures 1 and 2. Framed areas were cropped and demonstrated in Figure 1C and Figure 2B. B. No significant oscillations in the levels of eIF4E in the prefrontal cortex, hippocampus, and cerebellum. Representative western blots are shown on the left. Full length western images are shown to the right. *n* = 3 mice/group.
**Figure S4** (Related to Figure 4). Results from several control experiments before the main study. A. Total exploration time for Object 1 and Object 2 was not different between hand scoring and ANY‐maze scoring. Naïve WT mice (n = 6) were exposed to Object 1 (water bottle) or Object 2 (wooden cube) in a 10 min session. Time animals spent investigating the object was measured by both hand scoring and the ANY‐maze system. *n.s*., not significant. B. Total exploration time for Object 1 and Object 2 was not different among mice of different genotypes. Naïve mice were exposed to Object 1 (water bottle) or Object 2 (wooden cube) in a 10 min session. Time animals spent investigating the object was measured. n = 6 for each genotype. *n.s*., not significant. C. Diurnal variations of novel object recognition (NOR) memory are similar between male and female mice. On the left a bar graph indicates the discrimination index, which was calculated as (Time _novel_‐Time _familiar_)/ (Time _novel_ + Time _familiar_). On the right a bar graph indicates the distance travelled in the NOR test. *n* = 4–6 mice/group. Data are presented as individual values and mean ± SEM. *P < 0.05, ****P < 0.0001, *n.s*., not significant. Note that no difference was found between male and female mice. Significant difference was found between day (ZT 6) and night (ZT 18). D. Diurnal variations of object location memory (OLM) are similar between male and female mice. On the left a bar graph indicates the discrimination index, which was calculated as (Time _novel_‐Time _familiar_)/ (Time _novel_ + Time _familiar_). On the right a bar graph indicates the distance travelled in the NOR test. *n* = 4–6 mice/group. Data are presented as individual values and mean ± SEM. *P < 0.05, *n.s*., not significant. Note that significant difference was found between day (ZT 6) and night (ZT 18) but no difference was found between male and female mice.

## Data Availability

The data that support the findings of this study are available from the corresponding author upon reasonable request.

## References

[ejn15678-bib-0001] Aguilar‐Valles, A. , Haji, N. , De Gregorio, D. , Matta‐Camacho, E. , Eslamizade, M. J. , Popic, J. , Sharma, V. , Cao, R. , Rummel, C. , Tanti, A. , Wiebe, S. , Nuñez, N. , Comai, S. , Nadon, R. , Luheshi, G. , Mechawar, N. , Turecki, G. , Lacaille, J. C. , Gobbi, G. , & Sonenberg, N. (2018). Translational control of depression‐like behavior via phosphorylation of eukaryotic translation initiation factor 4E. Nature Communications, 9(1), 2459. 10.1038/s41467-018-04883-5 PMC6018502 29941989

[ejn15678-bib-0002] Amir, S. , & Stewart, J. (2009). Motivational modulation of rhythms of the expression of the clock protein PER2 in the limbic forebrain. Biological Psychiatry, 65(10), 829–834. 10.1016/j.biopsych.2008.12.019 19200536

[ejn15678-bib-0003] Amorim, I. S. , Kedia, S. , Kouloulia, S. , Simbriger, K. , Gantois, I. , Jafarnejad, S. M. , Li, Y. , Kampaite, A. , Pooters, T. , Romanò, N. , & Gkogkas, C. G. (2018). Loss of eIF4E phosphorylation engenders depression‐like behaviors via selective mRNA translation. The Journal of Neuroscience, 38(8), 2118–2133. 10.1523/JNEUROSCI.2673-17.2018 29367404 PMC5824745

[ejn15678-bib-0004] Bach, M. E. , Hawkins, R. D. , Osman, M. , Kandel, E. R. , & Mayford, M. (1995). Impairment of spatial but not contextual memory in CaMKII mutant mice with a selective loss of hippocampal LTP in the range of the theta frequency. Cell, 81(6), 905–915. 10.1016/0092-8674(95)90010-1 7781067

[ejn15678-bib-0005] Cain, S. W. , Chalmers, J. A. , & Ralph, M. R. (2012). Circadian modulation of passive avoidance is not eliminated in arrhythmic hamsters with suprachiasmatic nucleus lesions. Behavioural Brain Research, 230(1), 288–290. 10.1016/j.bbr.2012.02.022 22366268

[ejn15678-bib-0006] Cain, S. W. , & Ralph, M. R. (2009). Circadian modulation of conditioned place avoidance in hamsters does not require the suprachiasmatic nucleus. Neurobiology of Learning and Memory, 91(1), 81–84. 10.1016/j.nlm.2008.10.005 19013252

[ejn15678-bib-0007] Cao, R. , Butcher, G. Q. , Karelina, K. , Arthur, J. S. , & Obrietan, K. (2013). Mitogen‐ and stress‐activated protein kinase 1 modulates photic entrainment of the suprachiasmatic circadian clock. The European Journal of Neuroscience, 37, 130–140. 10.1111/ejn.12028 23127194 PMC3575747

[ejn15678-bib-0008] Cao, R. , Gkogkas, C. G. , de Zavalia, N. , Blum, I. D. , Yanagiya, A. , Tsukumo, Y. , Xu, H. , Lee, C. , Storch, K. F. , Liu, A. C. , Amir, S. , & Sonenberg, N. (2015). Light‐regulated translational control of circadian behavior by eIF4E phosphorylation. Nature Neuroscience, 18(6), 855–862. 10.1038/nn.4010 25915475 PMC4446158

[ejn15678-bib-0009] Chaudhury, D. , & Colwell, C. S. (2002). Circadian modulation of learning and memory in fear‐conditioned mice. Behavioural Brain Research, 133, 95–108. 10.1016/S0166-4328(01)00471-5 12048177

[ejn15678-bib-0010] Chaudhury, D. , Wang, L. M. , & Colwell, C. S. (2005). Circadian regulation of hippocampal long‐term potentiation. Journal of Biological Rhythms, 20(3), 225–236. 10.1177/0748730405276352 15851529 PMC2581477

[ejn15678-bib-0011] Costa‐Mattioli, M. , Sossin, W. S. , Klann, E. , & Sonenberg, N. (2009). Translational control of long‐lasting synaptic plasticity and memory. Neuron, 61, 10–26. 10.1016/j.neuron.2008.10.055 19146809 PMC5154738

[ejn15678-bib-0048] Dolci, C. , Montaruli, A. , Roveda, E. , Barajon, I. , Vizzotto, L. , Grassi Zucconi, G. , & Carandente, F. (2003). Circadian variations in expression of the trkB receptor in adult rat hippocampus. Brain Research, 994(1), 67–72. 10.1016/j.brainres.2003.09.018 14642449

[ejn15678-bib-0012] Dubruille, R. , & Emery, P. (2008). A plastic clock: How circadian rhythms respond to environmental cues in Drosophila. Molecular Neurobiology, 38, 129–145. 10.1007/s12035-008-8035-y 18751931

[ejn15678-bib-0051] Eckel‐Mahan, K. L. (2012). Circadian Oscillations within the Hippocampus Support Memory Formation and Persistence. Frontiers in Molecular Neuroscience, 5, 46. 10.3389/fnmol.2012.00046 22529773 PMC3328119

[ejn15678-bib-0013] Eckel‐Mahan, K. L. , Phan, T. , Han, S. , Wang, H. , Chan, G. C. , Scheiner, Z. S. , & Storm, D. R. (2008). Circadian oscillation of hippocampal MAPK activity and cAmp: Implications for memory persistence. Nature Neuroscience, 11(9), 1074–1082. 10.1038/nn.2174 19160506 PMC2772165

[ejn15678-bib-0014] Fernandez, F. , Lu, D. , Ha, P. , Costacurta, P. , Chavez, R. , Heller, H. C. , & Ruby, N. F. (2014). Circadian rhythm. Dysrhythmia in the suprachiasmatic nucleus inhibits memory processing. Science, 346(6211), 854–857. 10.1126/science.1259652 25395537 PMC4459503

[ejn15678-bib-0015] Furic, L. , Rong, L. , Larsson, O. , Koumakpayi, I. H. , Yoshida, K. , Brueschke, A. , Petroulakis, E. , Robichaud, N. , Pollak, M. , Gaboury, L. A. , Pandolfi, P. P. , Saad, F. , & Sonenberg, N. (2010). eIF4E phosphorylation promotes tumorigenesis and is associated with prostate cancer progression. Proceedings of the National Academy of Sciences of the United States of America, 107(32), 14134–14139. 10.1073/pnas.1005320107 20679199 PMC2922605

[ejn15678-bib-0016] Genheden, M. , Kenney, J. W. , Johnston, H. E. , Manousopoulou, A. , Garbis, S. D. , & Proud, C. G. (2015). BDNF stimulation of protein synthesis in cortical neurons requires the MAP kinase‐interacting kinase MNK1. The Journal of Neuroscience, 35, 972–984. 10.1523/JNEUROSCI.2641-14.2015 25609615 PMC4300335

[ejn15678-bib-0017] Gerstner, J. R. , & Yin, J. C. (2010). Circadian rhythms and memory formation. Nature Reviews. Neuroscience, 11(8), 577–588. 10.1038/nrn2881 20648063 PMC6544049

[ejn15678-bib-0019] Gkogkas, C. G. , Khoutorsky, A. , Cao, R. , Jafarnejad, S. M. , Prager‐Khoutorsky, M. , Giannakas, N. , Kaminari, A. , Fragkouli, A. , Nader, K. , Price, T. J. , Konicek, B. W. , Graff, J. R. , Tzinia, A. K. , Lacaille, J. C. , & Sonenberg, N. (2014). Pharmacogenetic inhibition of eIF4E‐dependent Mmp9 mRNA translation reverses fragile X syndrome‐like phenotypes. Cell Reports, 9(5), 1742–1755. 10.1016/j.celrep.2014.10.064 25466251 PMC4294557

[ejn15678-bib-0020] Kandel, E. R. , Dudai, Y. , & Mayford, M. R. (2014). The molecular and systems biology of memory. Cell, 157, 163–186. 10.1016/j.cell.2014.03.001 24679534

[ejn15678-bib-0021] Karatsoreos, I. N. , & Silver, R. (2007). Minireview: The neuroendocrinology of the suprachiasmatic nucleus as a conductor of body time in mammals. Endocrinology, 148(12), 5640–5647. 10.1210/en.2007-1083 17901227 PMC3423957

[ejn15678-bib-0022] Krishnan, H. C. , & Lyons, L. C. (2015). Synchrony and desynchrony in circadian clocks: Impacts on learning and memory. Learning & Memory, 22(9), 426–437. 10.1101/lm.038877.115 26286653 PMC4561405

[ejn15678-bib-0023] Martino, T. A. , & Harrington, M. E. (2020). The time for circadian medicine. Journal of Biological Rhythms, 35(5), 419–420. 10.1177/0748730420946501 32746705

[ejn15678-bib-0024] McMartin, L. , Kiraly, M. , Heller, H. C. , Madison, D. V. , & Ruby, N. F. (2021). Disruption of circadian timing increases synaptic inhibition and reduces cholinergic responsiveness in the dentate gyrus. Hippocampus, 31, 422–434. 10.1002/hipo.23301 33439521 PMC8048473

[ejn15678-bib-0025] Mintz, E. M. , Marvel, C. L. , Gillespie, C. F. , Price, K. M. , & Albers, H. E. (1999). Activation of NMDA receptors in the suprachiasmatic nucleus produces light‐like phase shifts of the circadian clock in vivo. The Journal of Neuroscience, 19(12), 5124–5130. 10.1523/JNEUROSCI.19-12-05124.1999 10366645 PMC6782653

[ejn15678-bib-0026] Moy, J. K. , Khoutorsky, A. , Asiedu, M. N. , Black, B. J. , Kuhn, J. L. , Barragán‐Iglesias, P. , Megat, S. , Burton, M. D. , Burgos‐Vega, C. C. , Melemedjian, O. K. , Boitano, S. , Vagner, J. , Gkogkas, C. G. , Pancrazio, J. J. , Mogil, J. S. , Dussor, G. , Sonenberg, N. , & Price, T. J. (2017). The MNK‐eIF4E signaling Axis contributes to injury‐induced nociceptive plasticity and the development of chronic pain. The Journal of Neuroscience, 37(31), 7481–7499. 10.1523/JNEUROSCI.0220-17.2017 28674170 PMC5546114

[ejn15678-bib-0027] Nitabach, M. N. , & Taghert, P. H. (2008). Organization of the Drosophila circadian control circuit. Current Biology, 18, R84–R93. 10.1016/j.cub.2007.11.061 18211849

[ejn15678-bib-0028] Panda, S. (2016). Circadian physiology of metabolism. Science, 354(6315), 1008–1015. 10.1126/science.aah4967 27885007 PMC7261592

[ejn15678-bib-0029] Paul, M. J. , & Schwartz, W. J. (2007). On the chronobiology of cohabitation. Cold Spring Harbor Symposia on Quantitative Biology, 72(1), 615–621. 10.1101/sqb.2007.72.042 18419321

[ejn15678-bib-0030] Phan, T. X. , Chan, G. C. , Sindreu, C. B. , Eckel‐Mahan, K. L. , & Storm, D. R. (2011). The diurnal oscillation of MAP (mitogen‐activated protein) kinase and adenylyl cyclase activities in the hippocampus depends on the suprachiasmatic nucleus. The Journal of Neuroscience, 31(29), 10640–10647. 10.1523/JNEUROSCI.6535-10.2011 21775607 PMC3146036

[ejn15678-bib-0031] Prosser, R. A. , McArthur, A. J. , & Gillette, M. U. (1989). cGMP induces phase shifts of a mammalian circadian pacemaker at night, in antiphase to cAMP effects. Proceedings of the National Academy of Sciences of the United States of America, 86(17), 6812–6815. 10.1073/pnas.86.17.6812 2549549 PMC297936

[ejn15678-bib-0032] Proud, C. G. (2007). Signalling to translation: How signal transduction pathways control the protein synthetic machinery. The Biochemical Journal, 403, 217–234. 10.1042/BJ20070024 17376031

[ejn15678-bib-0033] Reppert, S. M. , & Weaver, D. R. (2002). Coordination of circadian timing in mammals. Nature, 418(6901), 935–941. 10.1038/nature00965 12198538

[ejn15678-bib-0034] Richter, J. D. , & Sonenberg, N. (2005). Regulation of cap‐dependent translation by eIF4E inhibitory proteins. Nature, 433(7025), 477–480. 10.1038/nature03205 15690031

[ejn15678-bib-0035] Ruby, N. F. (2021). Suppression of circadian timing and its impact on the Hippocampus. Frontiers in Neuroscience, 15, 642376. 10.3389/fnins.2021.642376 33897354 PMC8060574

[ejn15678-bib-0036] Silver, R. , & Kriegsfeld, L. J. (2014). Circadian rhythms have broad implications for understanding brain and behavior. The European Journal of Neuroscience, 39(11), 1866–1880. 10.1111/ejn.12593 24799154 PMC4385795

[ejn15678-bib-0037] Smarr, B. L. , Jennings, K. J. , Driscoll, J. R. , & Kriegsfeld, L. J. (2014). A time to remember: The role of circadian clocks in learning and memory. Behavioral Neuroscience, 128, 283–303. 10.1037/a0035963 24708297 PMC4385793

[ejn15678-bib-0050] Snider, K. H. , Dziema, H. , Aten, S. , Loeser, J. , Norona, F. E. , Hoyt, K. , & Obrietan, K. (2016). Modulation of learning and memory by the targeted deletion of the circadian clock gene Bmal1 in forebrain circuits. Behavioural Brain Research, 308, 222–235. 10.1016/j.bbr.2016.04.027 27091299 PMC5344043

[ejn15678-bib-0039] Tosini, G. , Baba, K. , Hwang, C. K. , & Iuvone, P. M. (2012). Melatonin: An underappreciated player in retinal physiology and pathophysiology. Experimental Eye Research, 103, 82–89. 10.1016/j.exer.2012.08.009 22960156 PMC3462291

[ejn15678-bib-0040] Ueda, T. , Watanabe‐Fukunaga, R. , Fukuyama, H. , Nagata, S. , & Fukunaga, R. (2004). Mnk2 and Mnk1 are essential for constitutive and inducible phosphorylation of eukaryotic initiation factor 4E but not for cell growth or development. Molecular and Cellular Biology, 24(15), 6539–6549. 10.1128/MCB.24.15.6539-6549.2004 15254222 PMC444855

[ejn15678-bib-0041] Vogel‐Ciernia, A. , & Wood, M. A. (2014). Examining object location and object recognition memory in mice. Current Protocols in Neuroscience, 69, 31–17. 10.1002/0471142301.ns0831s69 25297693 PMC4219523

[ejn15678-bib-0049] Wang, L. M.‐C. , Dragich, J. M. , Kudo, T. , Odom, I. H. , Welsh, D. K. , O'Dell, T. J. , & Colwell, C. S. (2009). Expression of the Circadian Clock Gene Period2 in the Hippocampus: Possible Implications for Synaptic Plasticity and Learned Behaviour. ASN Neuro, 1(3), e00012. 10.1042/an20090020 19570032 PMC2695588

[ejn15678-bib-0042] Wardlaw, S. M. , Phan, T. X. , Saraf, A. , Chen, X. , & Storm, D. R. (2014). Genetic disruption of the core circadian clock impairs hippocampus‐dependent memory. Learning & Memory, 21(8), 417–423. 10.1101/lm.035451.114 25034823 PMC4105720

[ejn15678-bib-0043] Waskiewicz, A. J. , Flynn, A. , Proud, C. G. , & Cooper, J. A. (1997). Mitogen‐activated protein kinases activate the serine/threonine kinases Mnk1 and Mnk2. The EMBO Journal, 16(8), 1909–1920. 10.1093/emboj/16.8.1909 9155017 PMC1169794

[ejn15678-bib-0044] Weil, Z. M. , & Nelson, R. J. (2014). Introduction to the special issue on circadian rhythms in behavioral neuroscience. Behavioral Neuroscience, 128(3), 237–239. 10.1037/a0036740 24886186

