# Supplementary material for: Circadian activities of the brain MNK‐eIF4E signalling axis contribute to diurnal rhythms of some cognitive functions
Source: Eur J Neurosci. 2022 May 7;56(1):3553–69. doi: 10.1111/ejn.15678 (PMC9477079; doi:10.1111/ejn.15678)
Supplement: Supplementary file 1 — Figure S1 (Related to Figure 1). Representative microscopic images indicating expression of phospho‐eIF4E in a coronal brain section. The following brain regions are magnified and displayed separately: MC (motor cortex), AIC (agranular insular cortex), mPFC (medial prefrontal cortex), En (endopiriform nucleus), DTT (dorsal tenia tecta), and Pir (piriform cortex). Scale bar in magnified images: 200 μm. Figure S2 (Related to Figure 1). Representative microscopic images indicating expression of phospho‐eIF4E in a coronal brain section. The following brain regions are magnified and displayed separately: MC (motor cortex), PSC (primary somatosensory cortex), Pir (piriform cortex), LOT (nucleus of the lateral olfactory tract), PVA (paraventricular thalamic nucleus, anterior part), GP (globus pallidus), CC (cingulate cortex), and CPu (caudate putamen). Scale bar: 200 μm. Figure S3. Western blotting images. A. Full length western blotting images of the blots shown in Figures 1 and 2. Framed areas were cropped and demonstrated in Figure 1C and Figure 2B. B. No significant oscillations in the levels of eIF4E in the prefrontal cortex, hippocampus, and cerebellum. Representative western blots are shown on the left. Full length western images are shown to the right. n = 3 mice/group. Figure S4 (Related to Figure 4). Results from several control experiments before the main study. A. Total exploration time for Object 1 and Object 2 was not different between hand scoring and ANY‐maze scoring. Naïve WT mice (n = 6) were exposed to Object 1 (water bottle) or Object 2 (wooden cube) in a 10 min session. Time animals spent investigating the object was measured by both hand scoring and the ANY‐maze system. n.s., not significant. B. Total exploration time for Object 1 and Object 2 was not different among mice of different genotypes. Naïve mice were exposed to Object 1 (water bottle) or Object 2 (wooden cube) in a 10 min session. Time animals spent investigating the object was measured. n [file EJN-56-3553-s001.docx]

**Supplementary Figures**

**
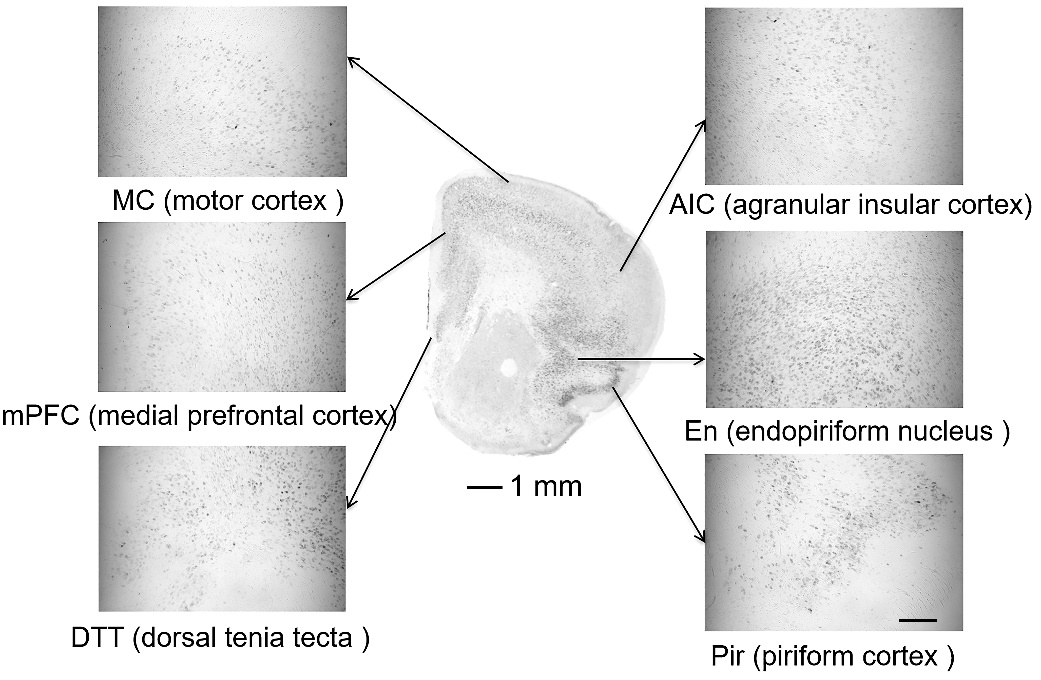
**

**Figure S1 (Related to Figure 1). Representative microscopic images indicating expression of phospho-eIF4E in a coronal brain section.** The following brain regions are magnified and displayed separately: MC (motor cortex), AIC (agranular insular cortex), mPFC (medial prefrontal cortex), En (endopiriform nucleus), DTT (dorsal tenia tecta), and Pir (piriform cortex). Scale bar in magnified images: 200 µm.

**
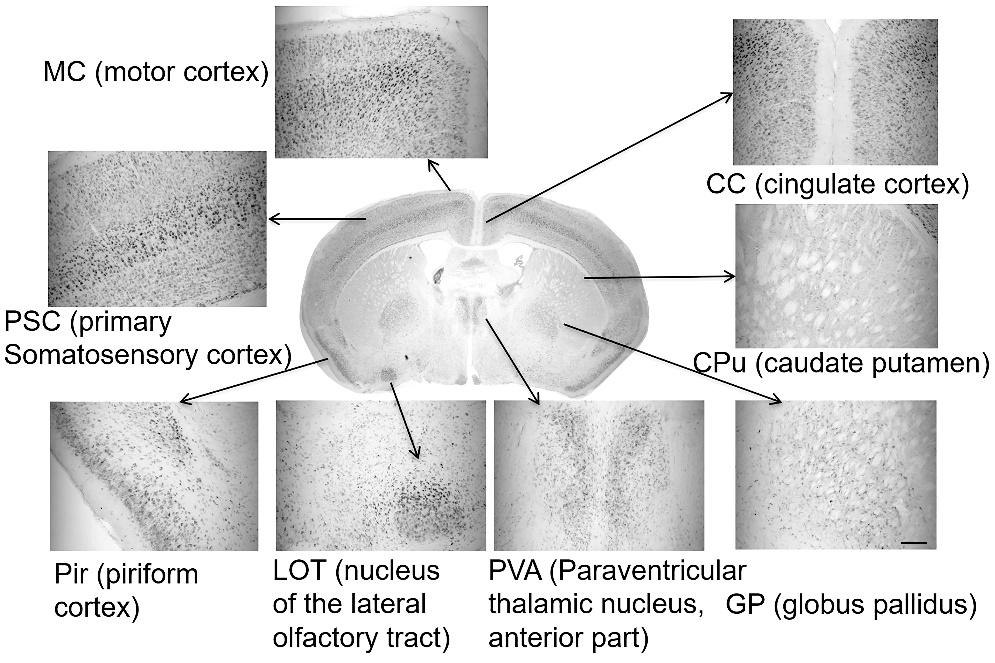
**

**Figure S2 (Related to Figure 1). Representative microscopic images indicating expression of phospho-eIF4E in a coronal brain section.** The following brain regions are magnified and displayed separately: MC (motor cortex), PSC (primary somatosensory cortex), Pir (piriform cortex), LOT (nucleus of the lateral olfactory tract), PVA (paraventricular thalamic nucleus, anterior part), GP (globus pallidus), CC (cingulate cortex), and CPu (caudate putamen). Scale bar: 200 µm.


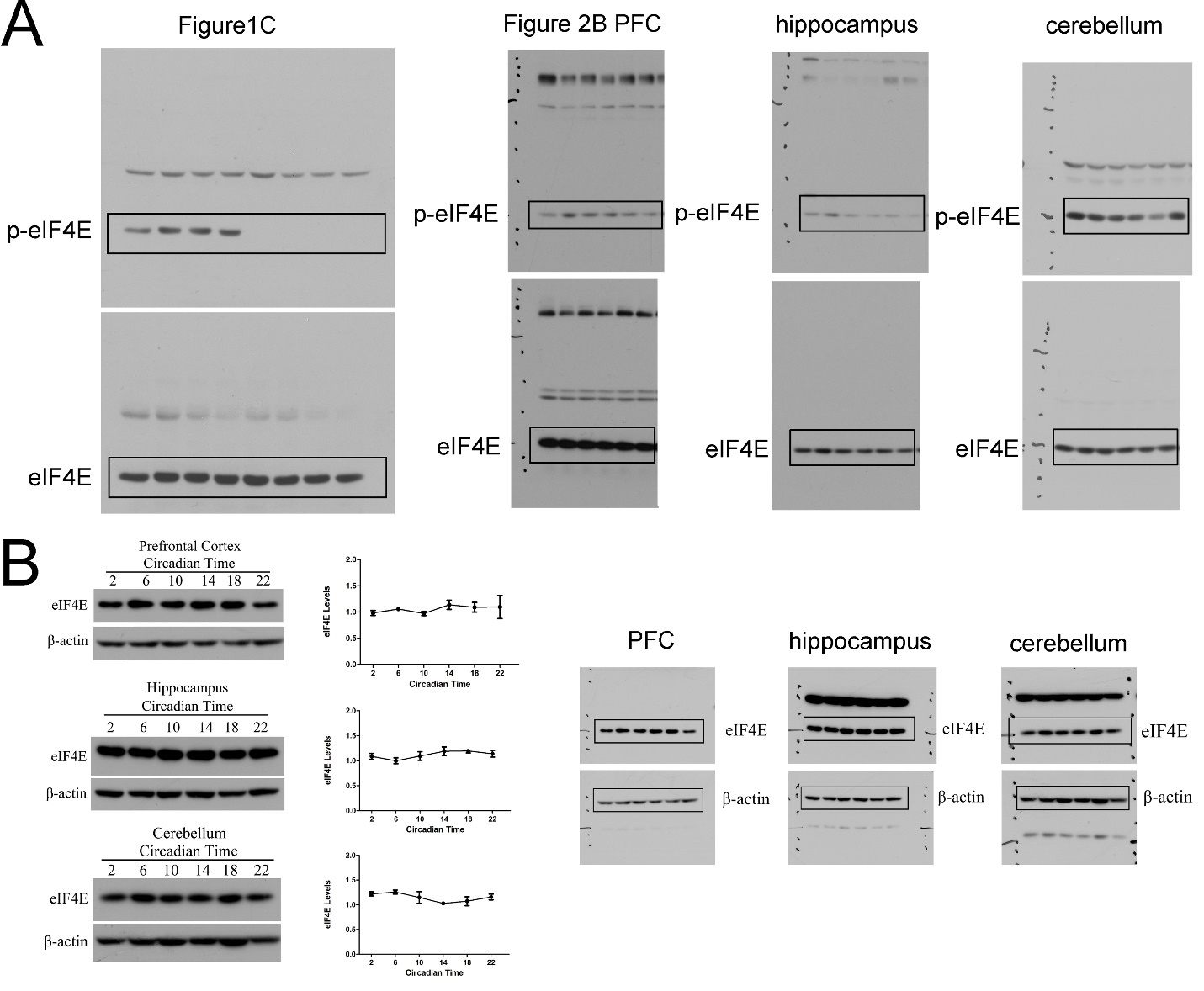


**Figure S3. Western blotting images. A.** Full length western blotting images of the blots shown in Figure 1 and 2. Framed areas were cropped and demonstrated in Figure 1C and Figure 2B. **B.** No significant oscillations in the levels of eIF4E in the prefrontal cortex, hippocampus, and cerebellum. Representative western blots are shown on the left. Full length western images are shown to the right. *n* = 3 mice/group.


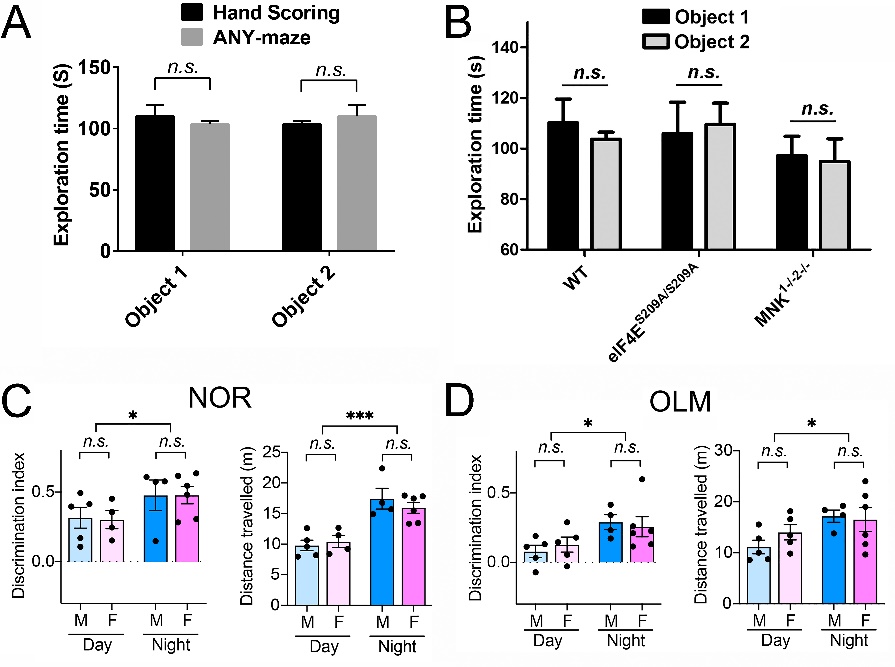


**Figure S4 (Related to Figure 4). Results from several control experiments before the main study.** A. Total exploration time for Object 1 and Object 2 was not different between hand scoring and ANY-maze scoring. Naïve WT mice (n=6) were exposed to Object 1 (water bottle) or Object 2 (wooden cube) in a 10 min session. Time animals spent investigating the object was measured by both hand scoring and the ANY-maze system. *n.s*., not significant. B. Total exploration time for Object 1 and Object 2 was not different among mice of different genotypes. Naïve mice were exposed to Object 1 (water bottle) or Object 2 (wooden cube) in a 10 min session. Time animals spent investigating the object was measured. n= 6 for each genotype. *n.s*., not significant. C. Diurnal variations of novel object recognition (NOR) memory are similar between male and female mice. On the left a bar graph indicates the discrimination index, which was calculated as (Time _novel_-Time _familiar_)/ (Time _novel_ + Time _familiar_). On the right a bar graph indicates the distance travelled in the NOR test. *n* = 4-6 mice/group. Data are presented as individual values and mean ± SEM. *P < 0.05, ****P < 0.0001, *n.s*., not significant. Note that no difference was found between male and female mice. Significant difference was found between day (ZT 6) and night (ZT 18). D. Diurnal variations of object location memory (OLM) are similar between male and female mice. On the left a bar graph indicates the discrimination index, which was calculated as (Time _novel_-Time _familiar_)/ (Time _novel_ + Time _familiar_). On the right a bar graph indicates the distance travelled in the NOR test. *n* = 4-6 mice/group. Data are presented as individual values and mean ± SEM. *P < 0.05, *n.s*., not significant. Note that significant difference was found between day (ZT 6) and night (ZT 18) but no difference was found between male and female mice.
